# Global burden and trends of high alcohol use-related injuries from 1990 to 2030: a comprehensive assessment of self-harm and interpersonal violence, transport injuries, and unintentional injuries using global burden of disease 2021

**DOI:** 10.3389/fpubh.2025.1675607

**Published:** 2025-10-16

**Authors:** Qiheng Yuan, Zhixuan Chen, Bianjin Sun, Chenzhi Zheng, Yutong Kang, Yongliang Lou, Meiqin Zheng

**Affiliations:** ^1^National Clinical Research Center for Ocular Diseases, Eye Hospital, Wenzhou Medical University, Wenzhou, China; ^2^Wenzhou Key Laboratory of Sanitary Microbiology, Key Laboratory of Laboratory Medicine, Ministry of Education, School of Laboratory Medicine and Life Science, Wenzhou Medical University, Wenzhou, China; ^3^Department of Clinical Laboratory, Shanghai Pulmonary Hospital, School of Medicine, Tongji University, Shanghai, China; ^4^Department of Clinical Laboratory, Beijing Ditan Hospital, Capital Medical University, Beijing, China

**Keywords:** high alcohol use, self-harm and interpersonal violence, transport injuries, unintentional injuries, GBD, SDI

## Abstract

**Background:**

High alcohol use (HAU) is a major global public health concern, contributing to injuries such as Self-harm and interpersonal violence (SIV), Transport injuries (TI), and Unintentional injuries (UII). However, comprehensive global assessments of HAU-related injury burden remain limited.

**Methods:**

Using data from Global Burden of Disease (GBD) 2021, we estimated HAU-related mortality and disability-adjusted life years (DALYs) from 1990 to 2021 across 204 countries and regions. We analyzed trends in Age-Standardized Disability-Adjusted Life Years Rate (ASDR) and Age-Standardized Mortality Rate (ASMR), examined Socio-demographic index (SDI) disparities, and employed age-period-cohort (APC) and Bayesian APC (BAPC) models for future projections. Frontier analysis identified countries with the greatest potential for burden reduction.

**Results:**

Despite the decline in ASDR and ASMR in overall global injuries, Low-middle SDI regions continue to experience increasing SIV and TI burdens (ASDR rose from 57.25 to 70.55; 25.08 to 30.8 respectively), while UII remains high in High-middle and High SDI countries (The ASDR were 57.94 and 59.12 respectively). Young adults and the elderly bear the greatest burden. BAPC projections indicate that China, India, and several high-burden nations will see further increases in DALYs and ASDR by 2030, highlighting the need for urgent interventions.

**Conclusion:**

Targeted policy measures, such as raising the legal drinking age, strengthening alcohol control for young people in Low and Low-middle-SDI regions, and enhancing older adults healthcare services in High-SDI regions, are essential to mitigate HAU-related injuries. Evidence-based, SDI-adapted strategies can significantly reduce this burden.

## Introduction

Alcohol is a toxic psychoactive substance that harms the body, damages the liver and brain, induces addiction, increases accident risk, and disrupts family and social relationships (1–5). It remains a major risk factor for global mortality and disease burden (6–8). In 2019, alcohol consumption contributed to 2,600,000 deaths annually, accounting for 4.7% of the global disease burden (9). Beyond its direct health effects, alcohol significantly impacts others, causing 298,000 deaths from alcohol-related road traffic accidents in 2019. Additionally, alcohol has been consistently identified as an important risk factor in interpersonal violence, suicide, and falls (10–12). From 1990 to 2017, the global per capita alcohol consumption rose from 7.1 to 11.2 liters and is projected to increase further (13), highlighting the urgent need to assess the injury burden associated with alcohol use.

Existing research primarily focuses on alcohol-related diseases such as cirrhosis, liver cancer, cardiovascular diseases, and neurological disorders (14–18), while studies on alcohol-related injuries remain limited. The Global Burden of Disease (GBD) 2021 study categorizes injuries attributable to High Alcohol Use (HAU) as a risk factor into three groups: Self-Harm and Interpersonal Violence (SIV), Transport Injuries (TI), and Unintentional Injuries (UII). TI is further divided into road transport injuries and other transport-related injuries, while UII includes falls, drowning, and similar incidents. Although existing studies have evaluated the correlation and relationship between alcohol-induced harm, road traffic injuries, suicide, interpersonal violence, drowning (19–23), they have generally been restricted to particular injury types, populations, or regions. A comprehensive, long-term global analysis covering the full spectrum of HAU-related injuries and their trends across different socio-demographic settings is still lacking.

This study fills this gap by using GBD 2021 data to provide, for the first time, a systematic global and regional assessment of mortality and Disability-Adjusted Life Years (DALYs) attributable to HAU-related injuries (SIV, TI, and UII) across 204 countries and territories from 1990 to 2021. By integrating the Socio-demographic Index (SDI), we further identify disparities in injury patterns, assess age- and sex-specific differences, and project future burdens in high-risk regions. The novelty of this study lies in its comprehensive scope and policy-oriented perspective, offering new insights into the global dynamics of alcohol-related injuries. These findings expand the current evidence base, highlight priority areas for intervention, and provide a scientific foundation for designing more effective, cost-efficient alcohol and injury prevention strategies.

## Methods

### Data sources

This study examined the impact of excessive alcohol consumption on injuries, SIV, TI, and UII using data from the GBD 2021 database. Notably, the database provides indicators for injuries and TI across individuals aged 5–95 years and above, whereas SIV and UII data are available only for those aged 15–95 years and above. Curated by the Institute for Health Metrics and Evaluation (IHME) (https://ghdx.healthdata.org/gbd-2021/sources), GBD 2021 offers extensive disease burden data for 204 countries, covering 371 diseases and injuries (24), 288 mortality causes (25), and 88 risk factors (8). By quantifying health losses across regions, time frames, and demographic groups, it supports efforts to enhance global health systems and reduce disparities. Within the GBD 2021 classification, injuries represent a primary category, while SIV, TI, and UII are classified as secondary subcategories.

This study utilized the Global Health Data Exchange (GHDx) query tool to retrieve alcohol-related indicators for injuries, SIV, TI, and UII from the GBD 2021 database, covering deaths and DALYs globally and by country from 1990 to 2021. DALYs, representing the sum of YLDs and years of life lost (YLLs) [24], quantify disease burden. YLDs are calculated as the number of affected individuals multiplied by disability weight, while YLLs correspond to the number of deaths multiplied by standard life expectancy loss.

### Calculation of age-standardized rates and EAPC for the burden of alcohol-related injuries, SIV, TI, and UII

We applied direct age standardization to determine the Age-Standardized Disability-Adjusted Life Years Rate (ASDR) and Age-Standardized Mortality Rate (ASMR) for alcohol-attributable injuries, SIV, TI, and UII. The application of this method is based on the fundamental assumption that the number of events (i.e., deaths or DALYs) within each age stratum follows a Poisson distribution (26). This statistical model is appropriate for count data of rare events, where each event can be considered independent and occurs with a low probability—assumptions that are generally valid for disease burden data such as mortality within specific age groups. By modeling the age-specific rates as a weighted sum of these independent Poisson random variables, the direct standardization method allows for the calculation of robust variance estimates and confidence intervals for the ASRs. To evaluate average trends over a specific period, we calculated the estimated annual percentage change (EAPC) in age-standardized rates (ASR) (27).

### Socio-demographic index (SDI)

As a composite indicator, the SDI quantifies a country's or region's level of socio-demographic development on a scale from 0 to 1, with higher values indicating more advanced social and economic development (28). In GBD 2021, countries and territories were grouped into five SDI strata: low (<0.47), low-middle (0.47–0.62), middle (0.62–0.71), high-middle (0.71–0.81), and high (>0.81) (29).

In our analysis, we examined the relationship between high alcohol use (HAU)-related injury burden and socio-economic development by stratifying results across these five SDI groups. Specifically, we compared ASDR and ASMR for overall HAU-related injuries and their subcategories SIV, TI, and UII) across SDI strata. This stratification enabled us to assess absolute and relative disparities in burden, evaluate temporal changes in ASDR and ASMR with respect to development level, and explore how socio-demographic advancement modifies the association between alcohol use and injury outcomes.

### Age-period-cohort (APC) model analysis

The APC model, widely applied in health and socioeconomic research, provides advantages over traditional methods. It estimates net and local drift, representing overall and specific temporal trends, respectively, while analyzing key temporal factors: age, period, and birth cohort (30–32). This study employed the APC model to examine ASMR trends of injuries, SIV, TI, and UII across different ages, periods, and cohorts. By isolating the effects of these factors, the model helps identify disease pattern changes associated with lifestyle shifts, medical advancements, or environmental influences.

This study employed the APC model, aligning age intervals with corresponding time periods, ensuring each 5-year age group matched a 5-year cycle. Data from the 2021 GBD database (1992–2021) were analyzed, with the target age range (5–95+ years) divided into 19 five-year categories (15–95+ years for SIV and UII). The study period was segmented into six 5-year intervals: 1992–1996, 1997–2001, 2002–2006, 2007–2011, 2012–2016, and 2017–2021. This framework also encompassed 22 overlapping 10-year birth cohorts, from 1892–1901 to 1997–2006.

The APC model estimated both overall and age-specific temporal trends. Net drift, representing the annual percentage change in incidence, captured the influence of calendar time and successive birth cohorts. Local drift, reflecting the annual percentage change in age-specific incidence, highlighted variations across age groups. The Wald χ^2^ test was used to assess the significance of these trends.

### Decomposition analysis

Decomposition analyses were conducted to determine key factors driving changes in alcohol-related injury burdens (SIV, TI, and UII) from 1990 to 2021. The study quantified the contributions of population growth, aging, and epidemiological transitions. Each factor's effect was isolated by holding the other two constant during evaluation (33).

### Cross-Country inequality analysis

The slope index of inequality (SII) and concentration index (CI) are commonly used to assess absolute and relative inequalities, respectively, by quantifying disparities in the burden of injuries, SIV, TI, and UII across countries (34). The SII, obtained through regression analysis, correlates a country's ASIR with its SDI rank, based on the population's cumulative midpoint. To account for heteroscedasticity, weighted regression models are employed. The CI, derived from the Lorenz curve, represents the association between cumulative ASDR, ASMR, and population distribution by SDI (35).

### Frontier analysis

Frontier analyses examined the relationship between alcohol-related injury burdens (SIV, TI, and UII) and sociodemographic development. The frontier, representing the minimum achievable burden, was modeled as a non-linear boundary dependent on a country's or region's development status. A non-parametric data envelopment analysis, based on established methodologies (36), was used to construct this frontier. The effective difference, defined as the gap between observed ASDR and ASMR rates and the frontier estimate, quantifies unrealized health improvements given the region's developmental stage.

### Prediction

The BAPC model predicted DALYs and ASDR for alcohol-related injuries, SIV, TI, and UII from 2022 to 2030. To enhance accuracy, it was combined with the Integrated Nested Laplace Approximation (INLA) framework, addressing mixing and convergence issues common in Bayesian methods using Markov Chain Monte Carlo (MCMC) sampling. The analysis employed R packages “BAPC” (v0.0.36) and “INLA” (v24.02.09), while statistical analyses and visualizations were conducted with Stata 16.0 and R 4.4.3.

### Statistical analysis

All statistical measures (absolute counts and standardized rates) are reported with 95% confidence intervals (CI) or uncertainty intervals (UI), determined through the 25th to 975th percentile range derived from 1,000 posterior simulations. Rate values were standardized per 100,000 individuals. Data analysis and visualization were performed using R v4.4.3.

## Results

### Trends in the burden of HAU-related injuries, SIV, TI, and UII in the world and SDI region

**Figure 1:**
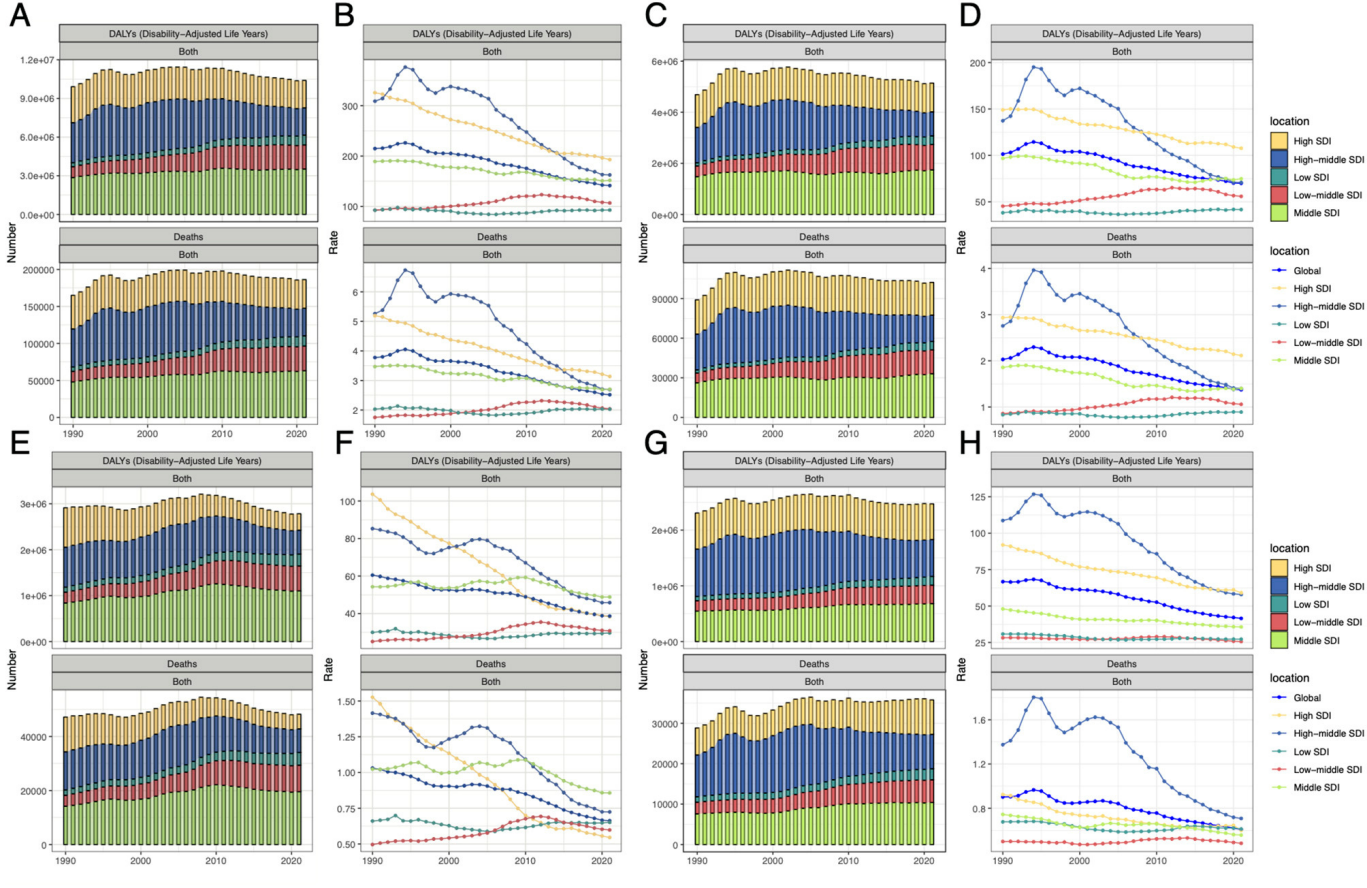
The DALYs and death numbers associated with HAU-related injuries, SIV, TI, and UII across five SDI regions from 1990 to 2021. **(A, C, E, G)** represent injuries, SIV, TI, and UII, respectively, while **(B, D, F, and H)** display their corresponding age-standardized rates, ASDR and ASMR. SDI, socio-demographic index; HAU, high alcohol use; SIV, self-harm and interpersonal violence; TI, transport injuries; UII, unintentional injuries; DALYs, disability-adjusted life years; ASDR, age-standardized disability-adjusted life years rate; ASMR, age-standardized mortality rate.

Figure 1A, Table 1, and Supplementary Table S1 indicate that by 2021, HAU-related injuries resulted in an increase in DALYs from 9,922,567 to 10,407,190 and Deaths from 165,284 to 186,476. While Middle-high and High SDI regions showed a decline in both DALYs and Deaths, Low, Low-middle, and Middle SDI regions experienced an increase. Over the past three decades, global ASDR and ASMR have steadily declined, though regional variations persist. Middle-SDI regions follow global trends, whereas middle-high and high-SDI regions—despite having a greater burden—have seen a downward trajectory (Table 1, Supplementary Table S1, Figure 1B). Notably, low and low-middle SDI regions, despite lower burdens than the global average, exhibit an increasing trend. SIV, TI, and UII trends generally align with global patterns, but some distinctions exist. For instance, SIV burden has risen sharply in low-middle SDI regions, whereas TI burden has declined significantly in High SDI countries (Figures 1C–F). In HAU-related UII, high-SDI regions exhibit ASMR values below the global trend (Figures 1G, H). These disparities in DALYs, Deaths, ASDR, and ASMR highlight the need for further investigation. EAPC, calculated using ASDR, estimates the annual percentage change in different burdens (Table 1). Globally, ASDR declined with an EAPC of −1.56 (95% UI: −1.67 to −1.44). However, low-middle SDI regions showed a positive EAPC of 0.83 (95% CI: 0.63–1.03). In SIV, both Low-middle SDI (EAPC: 0.83) and Low SDI (EAPC: 0.12, 95% UI: −0.04–0.28) regions exhibited an increasing trend. ASMR trends were similar, with a global EAPC of −1.54 (95% UI: −1.67 to −1.41), while Low-middle SDI regions had an EAPC of 0.86 (95% UI: 0.67–1.05). In SIV burden, Low SDI regions showed an EAPC of 0.05 (95% UI: −0.13–0.23) (Supplementary Table S1).

**Table 1:** Global and SDI trends of injuries, self-harm and interpersonal violence, transport injuries and unintentional injuries DALYs from 1990 to 2021.

**Cause**	**Characteristics**	**1990**		**2021**		**1990–2021**
		**DALYs cases No. (95% UI)**	**ASDR per 100,000 No. (95% UI)**	**DALYs cases No. (95% UI)**	**ASDR per 100,000 No. (95% UI)**	**EAPC No. (95% UI)**
Injuries	Global	9,922,567.11 (6,969,084.55–13,315,943.58)	214.82 (149.57–290.16)	10,407,190.05 (7,351,695.33–14,148,414.57)	141.53 (100.04–192.15)	−1.56 (−1.67−1.44)
	Low SDI	314,270.34 (143,474.5–460,420.4)	92.64 (41.41–135.83)	755,589.72 (476,065.79–1,071,369.51)	92.97 (58.33–132.09)	−0.08 (−0.23–0.06)
	Low-middle SDI	838,149.39 (496,113.52–1,194,616.97)	92.81 (54.14–133.41)	1,869,941.64 (1,273,585.28–2,596,990.07)	107.05 (72.41–149.23)	0.83 (0.63–1.03)
	Middle SDI	2,868,367.47 (2,022,314.58–3,826,472.03)	189.15 (133.22–253.68)	3,526,472.44 (2,558,871.66–4,700,618.68)	151.9 (110.1–202.36)	−0.84 (−0.89−0.78)
	High–middle SDI	3,102,159.14 (2,143,289.75–4,212,584.57)	308.73 (212.24–420.57)	2,122,245.79 (1,427,796.88–2,980,803.19)	162.57 (110.07–225.71)	−2.68 (−3.07−2.3)
	High SDI	2,786,489.33 (1,891,686.87–3,785,649.43)	325.5 (222.02–440.67)	2,122,307.81 (1,352,056.7–3,062,357.67)	192.9 (126.01–271.59)	−1.77 (−1.83−1.72)
Self–harm and interpersonal violence	Global	4,695,522.15 (2,834,211.17–6,817,660.44)	127.95 (76.66–185.8)	5,147,422.02 (3,160,730.89–7,413,820.36)	88.27 (54.32–127.11)	−1.6 (−1.75−1.44)
	Low SDI	128,493.06 (52,055.68–216,055.3)	48.25 (19.82–80.7)	340,713.89 (184,961.25–535,816.04)	52.62 (28.39–82.74)	0.12 (−0.04–0.28)
	Low–middle SDI	416,711.96 (219,590.73–661,878.63)	57.25 (29.78–91.03)	992,793.14 (596,650.07–1,488,103.83)	70.55 (42.17–105.83)	1.11 (0.87–1.35)
	Middle SDI	1,481,349.6 (928,662.18–2,159,282.32)	122.17 (76.25–178.43)	1,743,541.8 (1,124,998.93–2,445,991.54)	94.39 (60.81–132.48)	−1.21 (−1.36−1.05)
	High–middle SDI	1,380,844.19 (818,742.28–2,010,060.35)	173.23 (102.42–252.06)	939,572.99 (550,251.8–1,368,447.36)	89.35 (52.42–130.45)	−3.11 (−3.6−2.61)
	High SDI	1,282,523.26 (729,588.69–1,894,834.18)	187.98 (107.37–277.22)	1,125,655.32 (608,125.47–1,698,035.82)	135.88 (75.07–204.32)	−1.12 (−1.17−1.07)
Transport injuries	Global	2,916,522.07 (1,485,626.24–4,615,871.48)	60.49 (30.75–95.93)	2,785,799.16 (1,369,235.45–4,557,229.01)	38.65 (19.01–63.12)	−1.36 (−1.48−1.24)
	Low SDI	109,787.91 (42,763.84–189,913.74)	29.98 (11.45–51.85)	259,015.56 (118,032.58–443,766.47)	29.65 (13.37–50.87)	−0.1 (−0.27–0.06)
	Low–middle SDI	234,250.77 (106,961.01–393,764.38)	25.08 (11.34–42.24)	544,776.27 (255,168.45–909,379.99)	30.8 (14.36–51.57)	1.04 (0.82–1.26)
	Middle SDI	840,845.76 (420,063.67–1,368,401.14)	54.27 (27.09–88.36)	1,102,032.4 (542,850.77–1,795,592.35)	48.81 (24.05–79.37)	−0.24 (−0.42−0.06)
	High–middle SDI	870,927.23 (443,876.97–1,368,625.45)	85.27 (43.42–134.25)	520,768.43 (254,171.28–851,856.47)	45.83 (22.43–74.16)	−1.94 (−2.26−1.62)
	High SDI	857,039.8 (440,715.92–1,320,854)	103.63 (53.4–159.55)	356,860 (172,946.56–574,173.26)	38.33 (18.84–60.84)	−3.45 (−3.61−3.29)
Unintentional injuries	Global	2,310,522.89 (878,995.99–4,302,044.2)	66.74 (24.73–125.02)	2,473,968.87 (935,920.81–4,666,387)	41.52 (15.71–78.26)	−1.7 (−1.82−1.59)
	Low SDI	75,989.36 (18,693.2–150,192.78)	30.8 (7.27–61.06)	155,860.27 (55,079.06–300,602.65)	27.27 (9.62–52.7)	−0.41 (−0.53−0.3)
	Low–middle SDI	187,186.66 (60,962.87–363,799.24)	28.19 (8.85–55.26)	332,372.24 (117,731.37–624,879.62)	25.64 (8.9–48.37)	−0.13 (−0.23−0.02)
	Middle SDI	546,172.11 (217,878.26–1,007,079.32)	48 (18.98–88.7)	680,898.23 (264,452.35–1,261,323.15)	35.66 (13.88–66.03)	−0.86 (−0.93−0.79)
	High–middle SDI	850,387.72 (321,348–1,582,331.77)	108.68 (40.48–202.84)	661,904.37 (243,872.95–1,270,274.41)	57.94 (21.83–110.98)	−2.62 (−2.97−2.27)
	High SDI	646,926.27 (228,925.52–1,246,763.86)	91.92 (33.09–176.66)	639,792.48 (228,859.82–1,260,987.3)	59.12 (21.74–115.87)	−1.41 (−1.46−1.36)

SDI, socio-demographic Index; 95% UI, 95% uncertainty intervals; DALYs, disability adjusted life years; ASDR, Age-standardized disability adjusted life years Rate; EAPC, estimated annual percentage change.

### Trends in ASDR and ASMR across SDI countries

**Figure 2:**
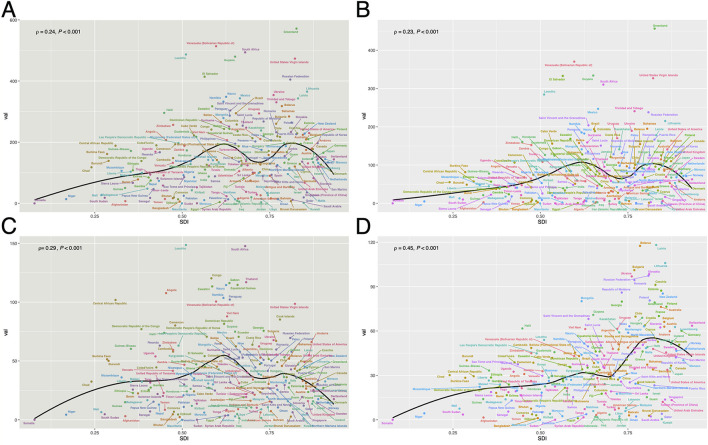
The relationship between ASDR and SDI for HAU-related injuries **(A)**, SIV **(B)**, TI **(C)**, and UII **(D)** across 204 countries in 2021. HAU, high alcohol use; SIV, self-harm and interpersonal violence; TI, transport injuries; UII, unintentional injuries; ASDR, age-standardized disability-adjusted life years rate; SDI, socio-demographic index.

Figure 2 illustrates the ASDR of injuries, SIV, TI and UII, as well as their relationship with SDI. Across all four categories, the burden initially rises with increasing SDI, then declines, followed by a secondary increase before ultimately decreasing. Countries with the highest overall injury burden include Greenland, South Africa, Lesotho, Guyana, and Venezuela (Figure 2A). Specifically, Greenland, Guyana, and Venezuela exhibit a greater burden from SIV (Figure 2B), while Lesotho faces more severe TI-related issues (Figure 2C). South Africa has high burdens in both SIV and TI, whereas Belarus and Latvia experience notably high UII burdens (Figure 2D). ASDR trends generally align with ASMR, though some discrepancies exist. For instance, Venezuela's ASDR exceeds its ASMR, suggesting a greater impact from disability-related injuries (Supplementary Figure S1).

### Global geographical distribution of ASDR and ASMR for HAU-related injuries, SIV, TI, UII

**Figure 3:**
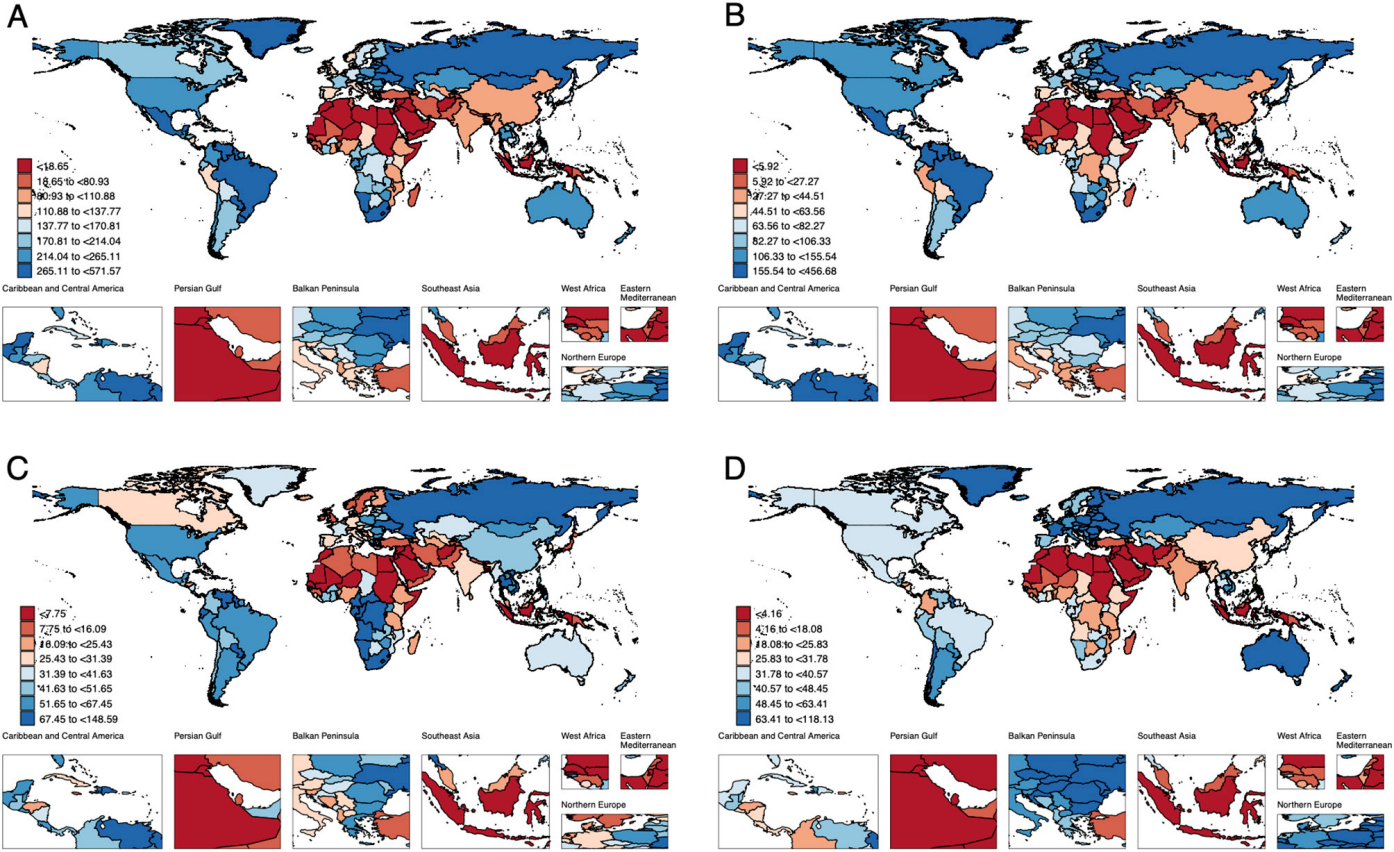
The geographic distribution of ASDR for HAU-related Injuries, SIV, TI, and UII across 204 countries and regions in 2021 [**(A)** Injuries, **(B)** SIV, **(C)** TI, **(D)** UII]. HAU, high alcohol use; SIV, self-harm and interpersonal violence; TI, transport injuries; UII, unintentional injuries; ASDR, age-standardized disability-adjusted life years rate.

Figure 3 presents the age-standardized mortality rate (ASMR) and age-standardized disability-adjusted life years (ASDR) for injuries, transport injuries, unintentional injuries, and self-harm and interpersonal violence (SIV) in 2021. ASDR data indicate that the overall injury burden is highest in North and South America, Central and Eastern Europe, northern Southeast Asia, and southern Africa (Figure 3A). When analyzed separately, SIV burden is more prominent in North and South America and Western Europe (Figure 3B), whereas transport and unintentional injuries are more concentrated in northern Southeast Asia and southern Africa (Figures 3C, D). In contrast, North Africa, the Persian Gulf region, and southern Southeast Asia exhibit relatively lower injury burdens (Figures 3A–D). ASMR trends largely align with ASDR patterns (Supplementary Figure S2).

### Temporal changes in the age distribution of DALYs for injuries, SIV, TI, and UII from 1992 to 2021

**Figure 4:**
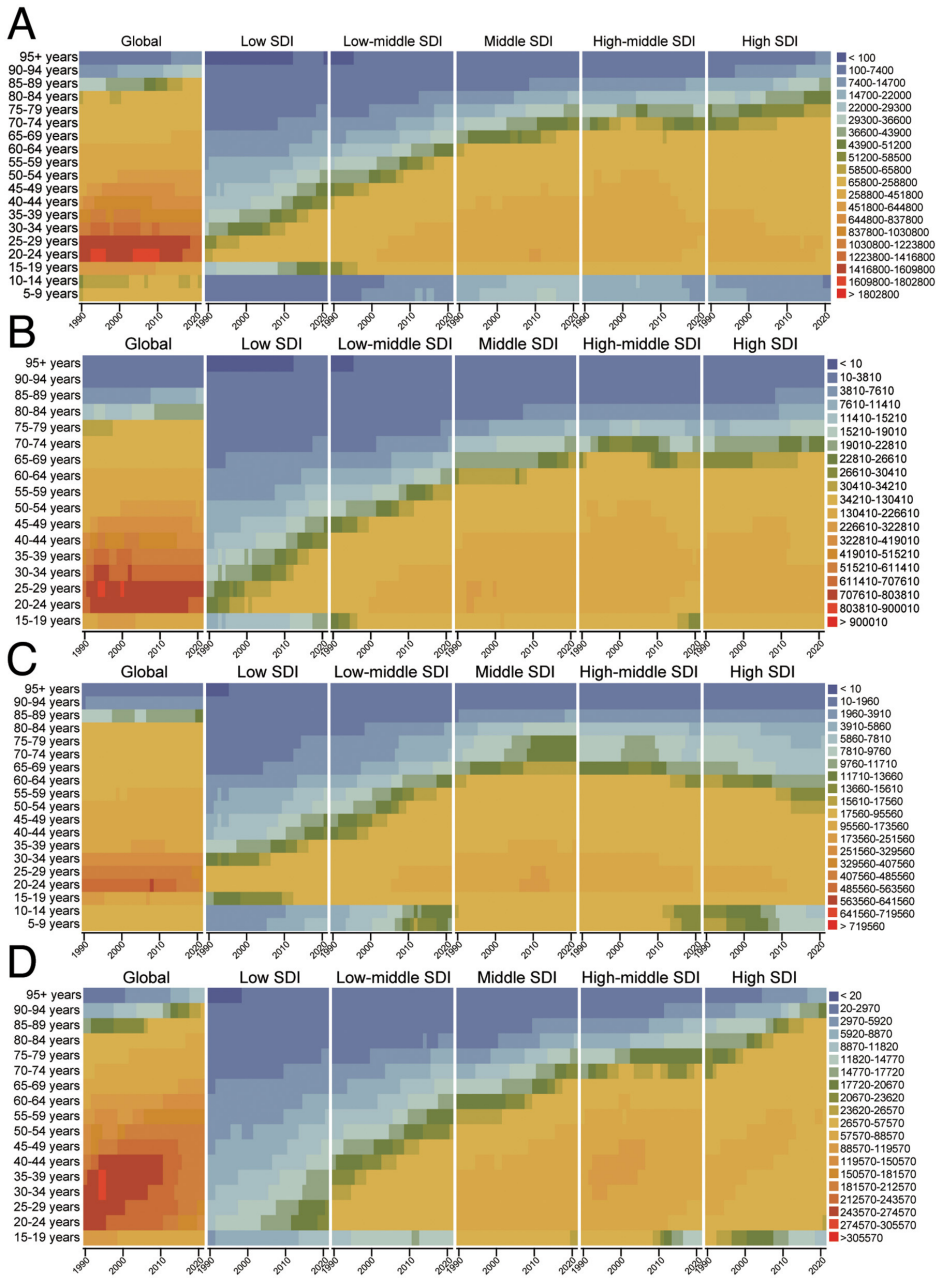
The temporal changes in the age distribution of DALYs for HAU-related Injuries, SIV, TI, and UII from 1990 to 2021 [**(A)** Injuries, **(B)** SIV, **(C)** TI, **(D)** UII]. HAU, high alcohol use; SIV, self-harm and interpersonal violence; TI, transport injuries; UII, unintentional injuries; ASDR, age-standardized disability-adjusted life years rate.

This study analyzed the temporal trends of four burden types across different age groups based on DALYs. Figure 4 indicates that HAU-related trends are similar across all four conditions. In terms of distribution, the burden is primarily concentrated among young adults aged 20–29, while UII remains prevalent between 20 and 45 years, with a significant burden also observed in individuals over 70. The figure further compares variations across different SDI regions, showing that DALYs for all injury types, except TI, increase with higher SDI levels (Figures 4A–D). Encouragingly, a comparison between 2021 and 1990 reveals that the overall DALY burden of these four injuries has remained stable over the past three decades, aligning with the findings of Figure 1.

### Effect of age, period, and birth cohort on the ASDR and ASMR of HAU-related injuries, SIV, TI and UII

**Figure 5:**
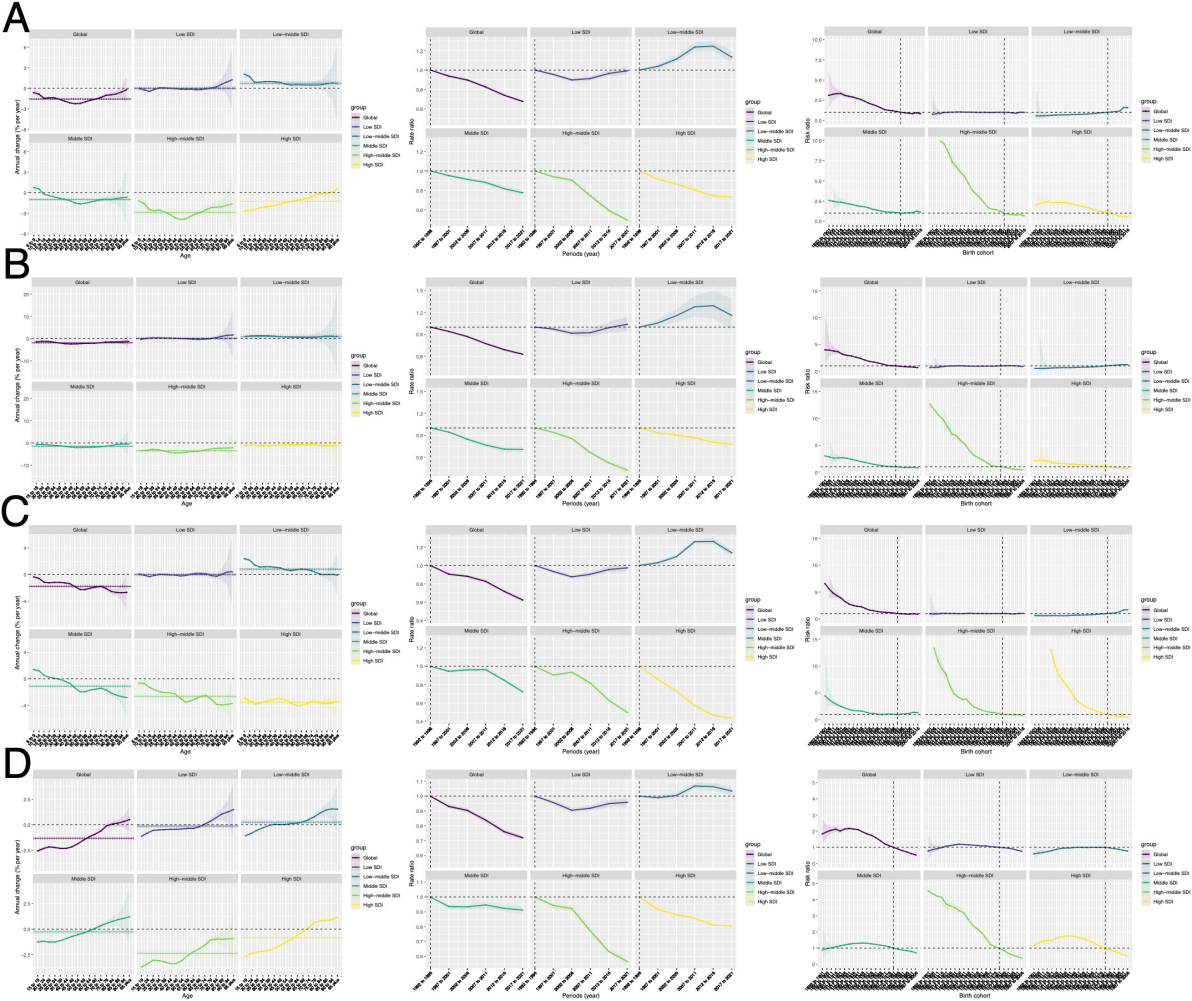
The effects of age, period, and birth cohort on ASDR for HAU-related Injuries, SIV, TI, and UII based on the APC model. Panels **(A)** represent the age, period, and cohort effects for Injuries, **(B)** for SIV, **(C)** for TI, and **(D)** for UII. APC, annual percentage change; HAU, high alcohol use; SIV, self-harm and interpersonal violence; TI, transport injuries; UII, unintentional injuries; ASDR, age-standardized disability-adjusted life years rate.

Figure 5 illustrates the influence of age, period, and birth cohort on HAU-related injuries, SIV, TI, and UII based on the APC model. For injuries, global ASDR age effects fluctuate but show negative annual changes across all age groups, with the highest risk observed in individuals aged 95 and above. In low-SDI regions, burden increases among those aged 70–74 and older, whereas in low-middle and middle-SDI regions, younger populations experience a rising burden. The period effect, using 1992–1996 as a baseline, reveals that only low-middle SDI regions exhibit risk ratios above the baseline, peaking in 2012–2016. Other SDI regions and global trends show a consistent decline. The birth cohort effect, with 1982–1991 as the reference, indicates that before this period, global risk remained above baseline, whereas later cohorts fell below it. This decline is driven by middle, high-middle, and high-SDI regions, particularly in high-middle SDI areas, where individuals born in 1892–1901 had nearly ten times the risk of those born in 1982–1991 (Figure 5A).

For SIV, global ASDR show negative annual changes across all age groups, while low-middle SDI regions exhibit positive changes. The period effect resembles that of injuries, except that in low-SDI regions, risk ratios exceeded the baseline from 2017–2021. The birth cohort effect also aligns with injury trends (Figure 5B). Similarly, TI results mirror injury trends in period and cohort effects, with age effects fluctuating across regions. However, most areas still show negative annual changes, except for young populations in low-middle and Middle-SDI regions and some older age groups in low-SDI regions, where rates are above zero (Figure 5C). For UII, the age effect shows a rising trend globally and across SDI regions, except for high-middle SDI areas, where all age groups exhibit negative annual changes. In other SDI regions, individuals over 70–74 years have positive annual change rates. The period and birth cohort effects are similar to injury trends (Figure 5D). The APC results analyzed using the ASMR data are generally similar to those of ASDR. It should be noted that in the birth cohort effect, the differences between different birth cohorts are smaller in the ASMR analysis results than in the ASDR results (Supplementary Figure S3).

### Decomposition analysis

**Figure 6:**
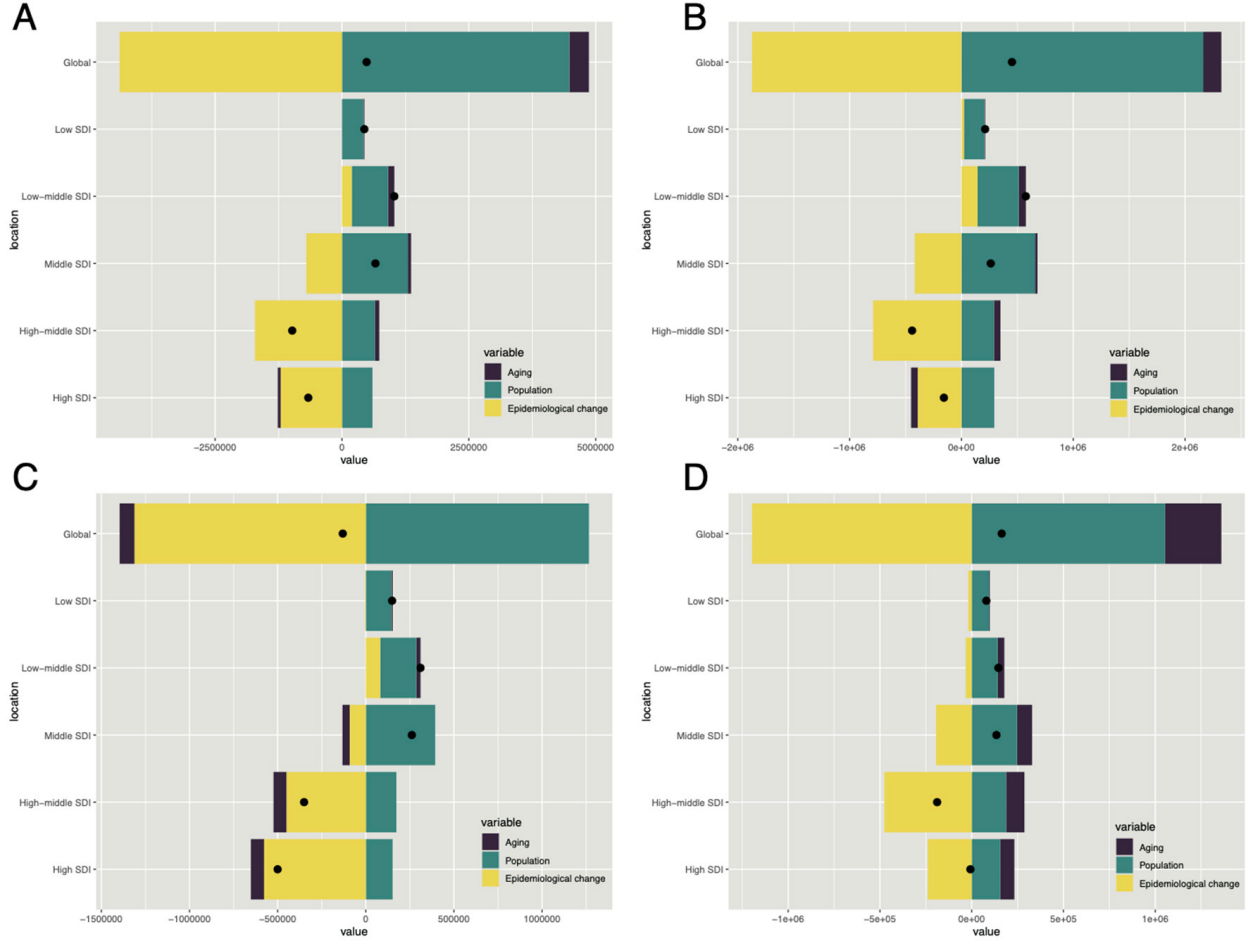
The relative contributions of aging, population dynamics, and epidemiological changes to ASDR variations in HAU-related Injuries, SIV, TI, and UII from 1990 to 2021, with comparisons between global data and five SDI regions. Black dots indicate the overall ASDR change during this period. Panels **(A–D)** corresponds to injuries, SIV, TI, and UII, respectively. HAU, high alcohol use; SIV, self-harm and interpersonal violence; TI, transport injuries; UII, unintentional injuries; ASDR, age-standardized disability-adjusted life years rate.

A decomposition analysis identified three key factors influencing changes in injury burden: epidemiological trends, population growth, and aging. Between 1990 and 2021, population growth was the primary driver of the global increase in injury-related deaths and DALYs, with a particularly pronounced effect on DALYs growth. This trend was consistent across different SDI regions, showing a positive correlation between population growth and overall injury burden (Figure 6A, Supplementary Figure S4A). From the perspectives of SIV, UII, and TI indicators, population growth remained the dominant contributing factor. However, aging played a more significant role in the increasing burden of SIV and UII, while its impact was less pronounced in TI. Notably, in the DALY decomposition analysis of TI, aging exhibited a negative correlation (Figures 6B–D). Across various SDI regions, these trends generally aligned with global patterns. Specifically, in SIV and TI, the contribution of aging to injury burden decreased as SDI increased, eventually becoming negative in some cases, a trend particularly evident in DALY results (Figure 6, Supplementary Figure S4). In contrast, UII exhibited an opposite pattern, where the influence of aging on injury burden intensified with higher SDI levels (Figure 6D, Supplementary Figure S4D).

### Healthy inequality analysis

**Figure 7:**
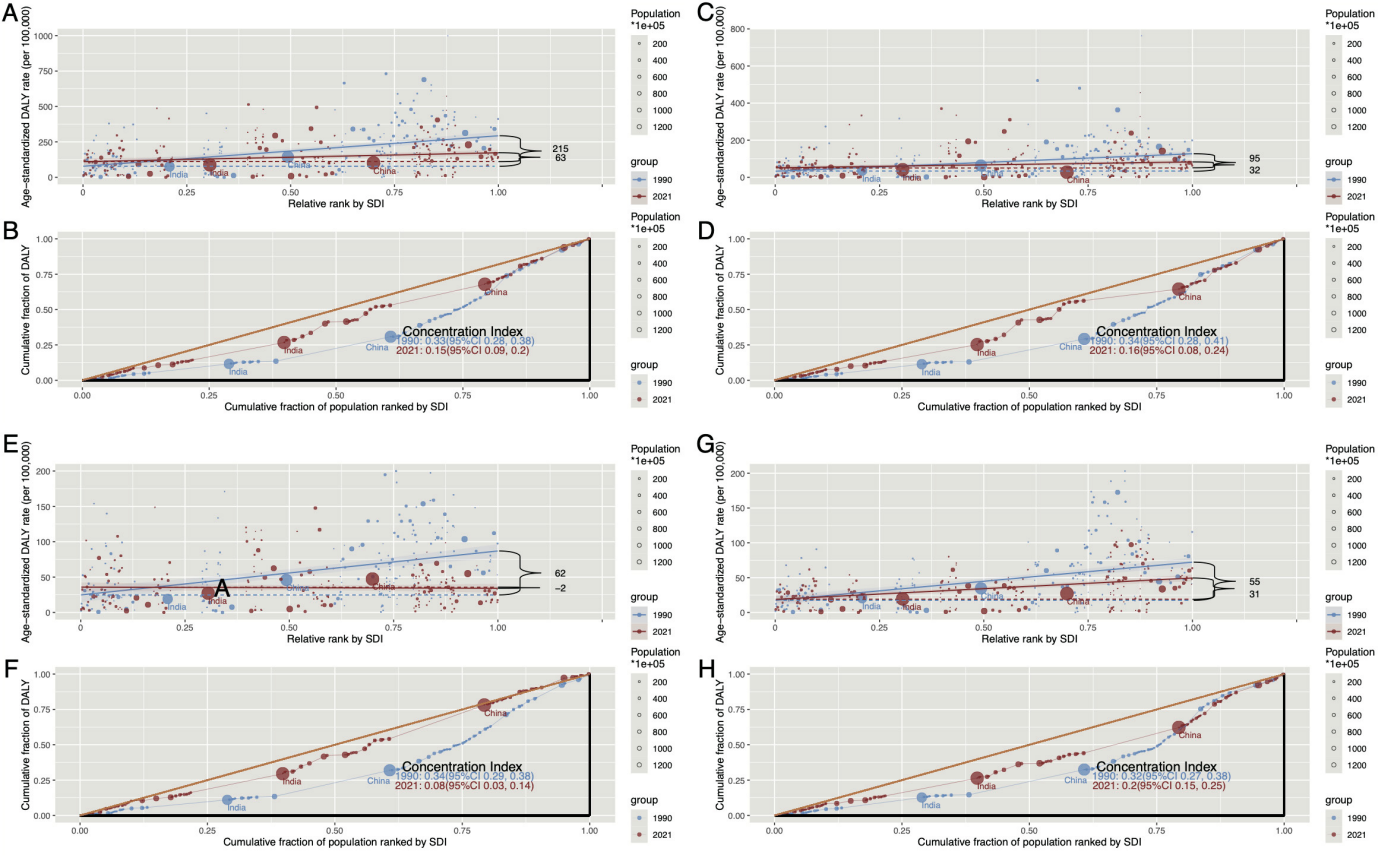
The Slope Index of Inequality (SII) and Concentration Index (CI) of ASDR for HAU-related Injuries, SIV, TI, and UII in 1990 and 2021 worldwide. **(A)** Represents the SII for Injuries, illustrating the relationship between SDI and ASIR across countries, with data point size proportional to population. **(B)** Displays the CI for Injuries, quantifying relative health disparities by measuring the area under the Lorenz curve, aligning ASIR distribution with the SDI-based population distribution. **(C, D)** Show the SII and CI for SIV, respectively, while **(E, F)** present the same for TI. Similarly, **(G, H)** depict the SII and CI for UII. SII, slope index of inequality; CI, concentration index; HAU, high alcohol use; SIV, self-harm and interpersonal violence; TI, transport injuries; UII, unintentional injuries; ASDR, age-standardized disability-adjusted life years rate; SDI, socio-demographic index.

Analysis of health inequality indicates that injuries attributable to HAU primarily affect high-SDI regions in terms of ASDR (Figures 7A, B). The SII reveals a narrowing national disparity, with values decreasing from 215.43 (95% CI: 158.84, 272.02) in 1990 to 62.93 (95% CI: 20.12, 105.74) in 2021 (Figure 7A). The concentration index (CL) suggests that while the burden remains higher among affluent populations, it has diminished over time, with values decline from 0.33 (95% CL: 0.28, 0.38) in 1990 to 0.15 (95% CL: 0.09, 0.20) in 2021 (Figure 7B). A similar trend is observed for ASMR (Supplementary Figures S5A, B). Regarding alcohol-related SIV, TI, and UII, both ASDR and ASMR indicate a pattern consistent with overall injury burden, with a higher concentration in high-SDI regions and wealthier populations, alongside a gradual reduction in disparities (Figure 7, Supplementary Figure S5). Notably, in 2021, TI burden was more prominent in low-SDI regions, as reflected by an ASDR SII of −1.65 (95% CL: −11.82, 8.50).

### Frontier analysis based on ASDR and ASMR

**Figure 8:**
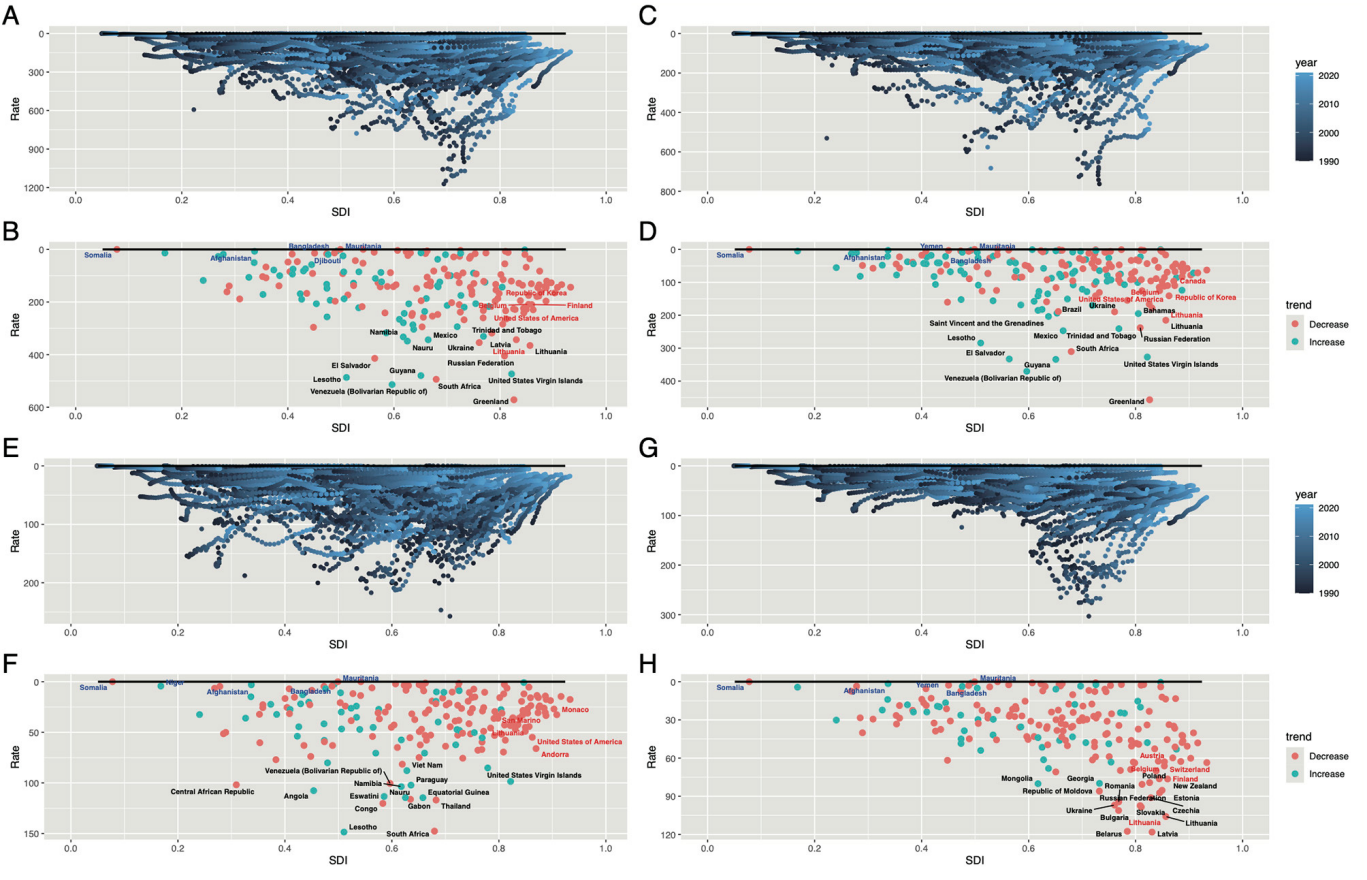
Frontier analysis of ASDR for HAU-related Injuries, SIV, TI, and UII based on SDI in 2021. The black boundary line represents the theoretically achievable ASDR given SDI, while each point denotes the actual ASDR of a specific country or region. Color gradients indicate data collection years, ranging from dark blue (1990) to light blue (2021). The 15 countries with the largest effective disparities—defined as the gap between observed and achievable ASDR—are marked in black. The five countries with the smallest disparities in Low-SDI regions (< 0.50) are highlighted in blue, whereas the country with the highest disparity in High-SDI regions (>0.85) is marked in red. Panels **(A, B)** correspond to Injuries, **(C, D)** to SIV, **(E, F)** to TI, and **(G, H)** to UII. HAU, high alcohol use; SIV, self-harm and interpersonal violence; TI, transport injuries; UII, unintentional injuries; ASDR, age-standardized disability-adjusted life years rate.

Figure 8 uses data from 1990 to 2021, this study integrates ASMR, ASDR, and SDI to analyze the relationship between HAU-related injury burden and national development levels. The frontier represents the theoretically achievable ASMR and ASDR at each SDI level, while the effective disparity quantifies the gap between a country's actual and attainable ASMR and ASDR based on its development stage. Overall, the injury burden follows trends similar to SIV and UII, with some High SDI countries exhibiting significant improvement potential (Figures 8A, C, G). However, frontier analysis of TI reveals heterogeneity, indicating substantial room for improvement in both High and Low SDI countries (Figure 8E). ASDR trends largely align with ASMR patterns (Supplementary Figures S6A, C, E, G).

By examining ASMR and ASDR, we identified 15 countries with the highest potential for reducing injuries, including Greenland, Lesotho, and South Africa for overall injuries; Greenland, Venezuela, and Guyana for SIV; Lesotho, South Africa, and Congo for TI; and Belarus, Latvia, and Lithuania for UII. Additionally, Somalia and Afghanistan, despite their Low SDI levels, were classified as frontier countries with minimal effective disparity, whereas Lithuania and the United States—High SDI nations—still exhibit substantial improvement potential (Supplementary Figures S8B, D, F, H).

### Age distribution of ASDR for HAU-related injuries, SIV, TI, UII in 2021 worldwide and in key regions

**Figure 9:**
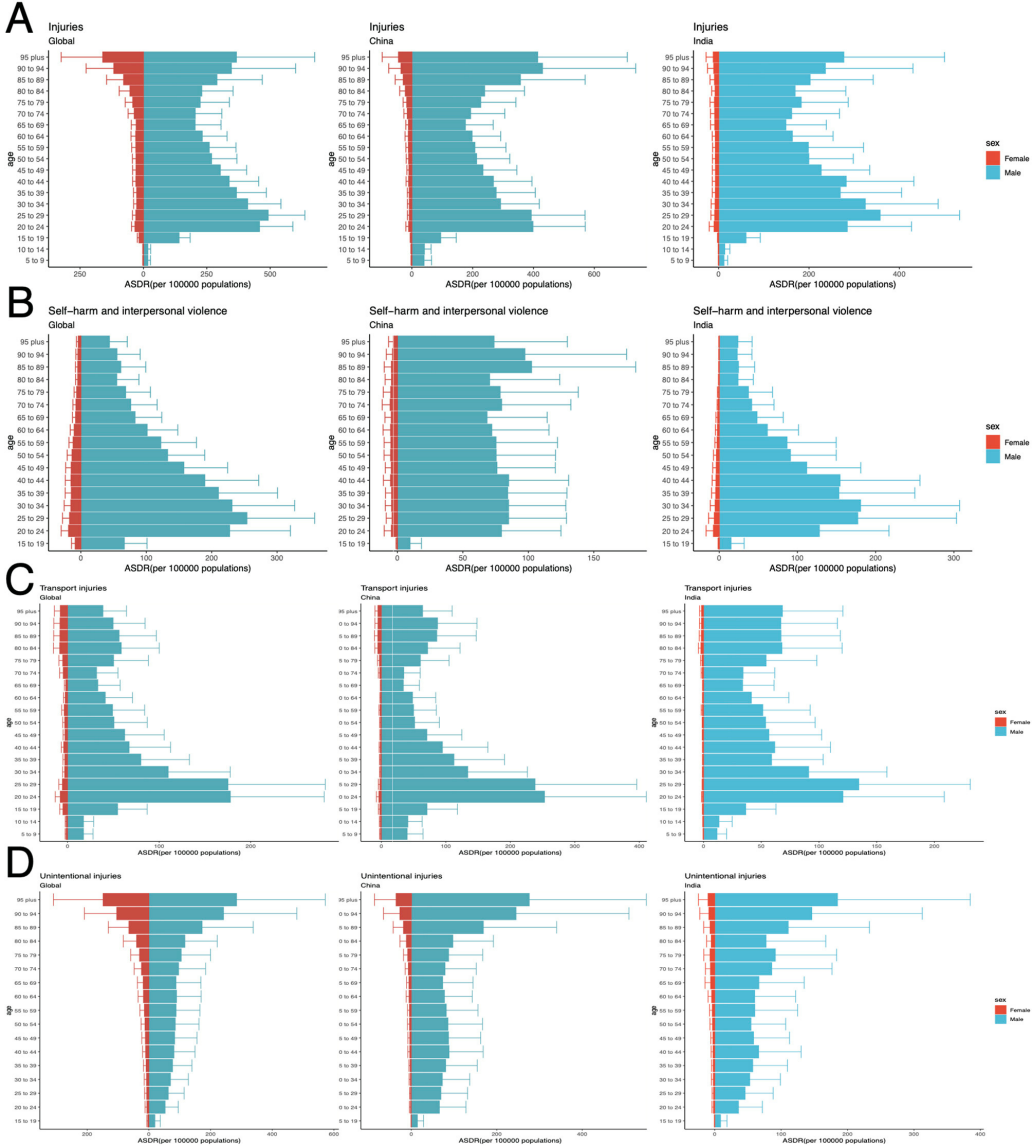
Age distribution of ASDR for HAU-related Injuries, SIV, TI, and UII in 2021 across the world, China, and India. Panels **(A–D)** corresponds to Injuries, SIV, TI, and UII, respectively. HAU, high alcohol use; SIV, self-harm and interpersonal violence; TI, transport injuries; UII, unintentional injuries; ASDR, age-standardized disability-adjusted life years rate.

This study analyzed the age distribution of ASDR burden in 2021 across selected countries. Results indicate that injury burden due to HAU was significantly higher in males than females, predominantly affecting individuals aged 20–40 and those over 90. The age distribution in China and India closely resembled global patterns (Figure 9A), whereas five countries with high improvement potential exhibited distinct trends, with DALY burden concentrated in the 20–40 age group, necessitating targeted attention (Supplementary Figures S7A–E). In SIV, global trends aligned with those of India and high-potential countries, with HAU-related burden primarily affecting males aged 20–40 (Figure 9B). Notably, in the United States Virgin Islands and Venezuela (Bolivarian Republic of), SIV burden was more pronounced among individuals aged 20–29, highlighting the need for further attention (Supplementary Figures S7F–J). Interestingly, China's distribution differed, with a relatively even burden across all age groups except for 15–19 years (Figure 9B). The TI burden followed a pattern similar to overall injury burden, primarily affecting young adults and individuals over 80 globally and across seven countries (Figure 9C, Supplementary Figures S7K–O). In contrast, UII burden exhibited a unique pattern, with global, Chinese, and Indian data showing a progressive increase in burden with age, disproportionately affecting males (Figure 9D). Notably, in high-potential countries, ASDR burden was concentrated in the 40–60 age group, emphasizing the critical role of this demographic in mitigating HAU-induced UII burden (Supplementary Figures S7P–T).

### Projected global burden of alcohol-related injuries, SIV, TI, and UII in 2030

**Figure 10:**
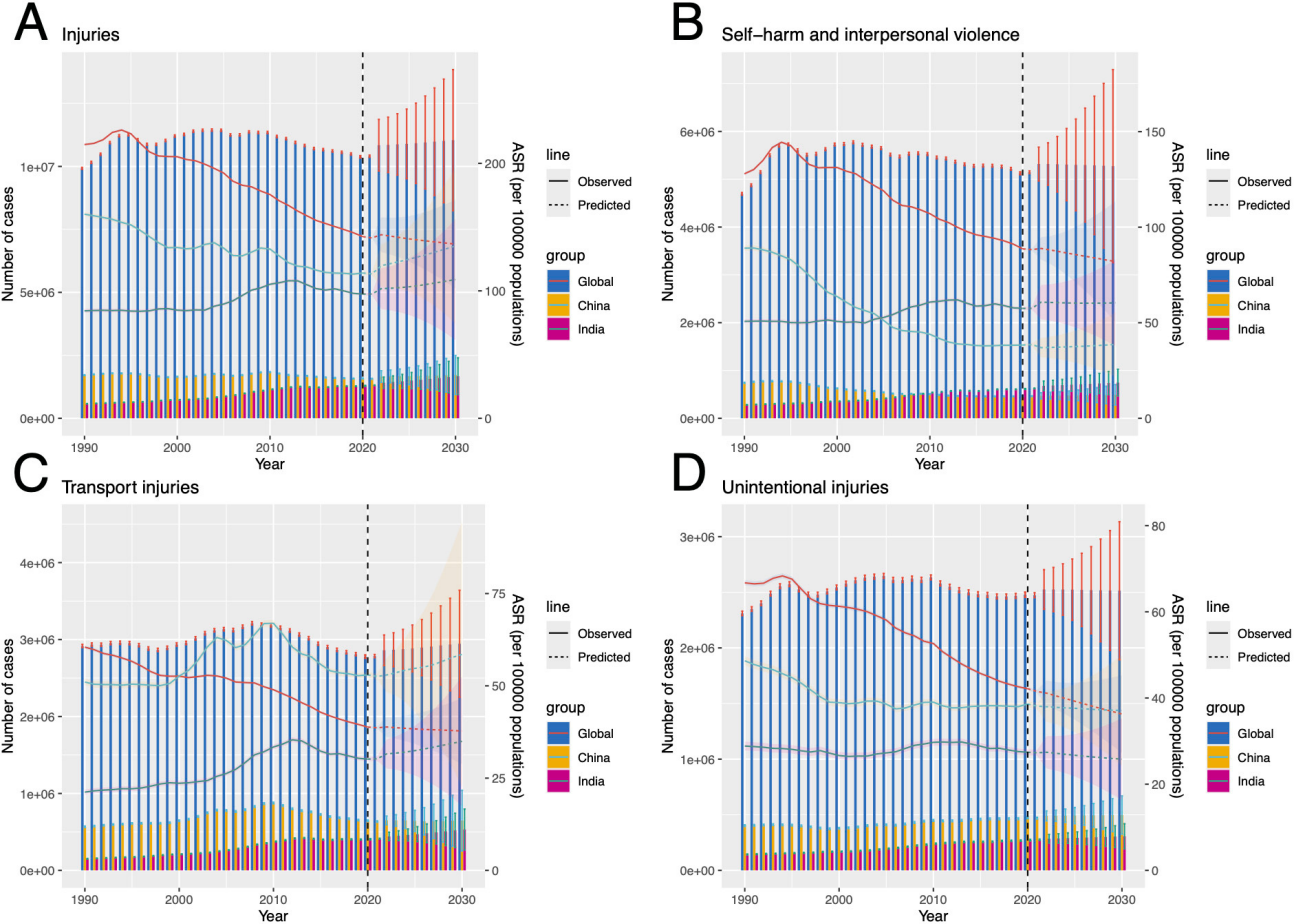
Projected DALYs and ASDR for HAU-related Injuries, SIV, TI, and UII globally, as well as in China and India, from 2022 to 2030 **(A–D)** corresponds to Injuries, SIV, TI, and UII. HAU, high alcohol use; SIV, self-harm and interpersonal violence; TI, transport injuries; UII, unintentional injuries; DALYs, disability-adjusted life years; ASDR, age-standardized disability-adjusted life years rate.

The study employed the BAPC model to project the burden of injuries, self-harm and interpersonal violence (SIV), and transport injuries (TI) caused by HAU globally and in major population centers (China and India) through 2030 (Figure 10). Based on DALYs and ASDR data, projections indicate that global injuries-related DALYs will increase from 10,407,170 (95% UI: 10,364,345–10,449,996) in 2021 to 11,038,162 (95% UI: 8,263,695–13,812,628) in 2030, while ASDR is expected to decline. In contrast, China and India are projected to experience an upward trend in both DALYs and ASDR (Figure 10A). For SIV, projections suggest a global decline in DALYs and ASDR, whereas in China and India, DALYs are expected to rise, particularly in India, increasing from 624,786 (95% UI: 616,470–633,103) to 747,058 (95% UI: 468,020–1,026,098), while ASDR remains relatively stable in both countries (Figure 10B). TI burden follows a pattern similar to injuries, with global DALYs projected to rise and ASDR to decline, whereas China and India are expected to experience an increase in ASDR (Figure 10C). Predictions for UII indicate a stable DALYs burden globally and in China and India, with ASDR expected to decrease (Figure 10D).

Based on ASMR and ASDR frontier analysis, the study further identified five countries with the highest improvement potential across four injury categories. In injuries, Lesotho, South Africa, and Venezuela are expected to see increasing DALYs and ASDR (Supplementary Figures S8A–E). For SIV, the burden is projected to rise in South Africa, the United States Virgin Islands, and Venezuela (Supplementary Figures S8F–J). TI projections are more concerning, with DALYs and ASDR increasing in Congo, Equatorial Guinea, Lesotho, and South Africa (Supplementary Figures S8K–O). Finally, UII projections indicate a continued increase in DALYs and ASDR in Belarus and Latvia (Supplementary Figures S8P–T).

## Discussion

Alcohol significantly impacts healthcare systems, with 5%−40% of all emergency department injury cases in 27 countries attributed to alcohol consumption (37). This imposes a substantial financial burden. In the United States, alcohol-related emergency visits surged by 61.6% from 3,080,214 in 2006 to 4,976,136 in 2014, while associated healthcare costs rose by 272%, from $4.1 billion to $15.3 billion (38). Similarly, in some developing countries, such as Sri Lanka, alcohol-related injury costs exceeded $380 million in 2015, accounting for nearly 50% of the country's total alcohol-related expenses (39). Evidence also links alcohol to interpersonal violence, self-harm, road traffic injuries, falls, drowning, and temperature-related harm. These injuries pose severe threats to both physical and mental health, disrupt social productivity, and cause extensive societal harm (20, 40–45). Given these consequences, analyzing the burden of alcohol-related injuries is imperative. This study employs DALYs, ASDR, and inequality analysis, along with decomposition and frontier analyses, to examine the global trends and regional disparities of HAU-related Injuries, SIV, TI, and UII. Additionally, APC and BAPC models are applied to investigate temporal dynamics and SDI-related variations, providing evidence-based predictions for future trends. The findings offer critical data to guide effective interventions. To our knowledge, this is the first study to comprehensively assess the burden of these four alcohol-related injuries across 204 countries and regions from 1990 to 2021, analyzing current trends, regional differences, and future projections at global, regional, and national levels.

This study highlights the significant relationship between HAU-related injury burden and SDI, revealing global disparities. While Deaths and DALYs have increased worldwide, ASDR and ASMR indicate an overall decline in HAU-related injuries. However, Low and Low-middle SDI regions continue to experience a rising burden, particularly for SIV and TI. This reflects the increasing awareness of alcohol-related harms in recent years among High-SDI countries, coupled with the implementation of government regulatory measures and the availability of well-developed healthcare systems, which together have contributed to the control of ASDR and ASMR. In contrast, Low-SDI countries often face limited healthcare resources, and in some cases, alcohol is regarded as an emerging market for growth and profit, thereby driving rising consumption in low- and middle-income countries and increasing the associated burden (46). Moreover, in low- and middle-SDI regions, the lack of road safety infrastructure and weak enforcement of traffic regulations further exacerbate the burden of traffic-related injuries compared with High-SDI countries (47).

In contrast, although High SDI countries show a declining trend, certain injury types, such as UII, remain prevalent in some High SDI nations. Frontier analysis further confirms that High SDI countries exhibit considerable room for improvement in UII burden reduction. These findings emphasize the heterogeneity of HAU-related injuries globally, underscoring the need for region-specific interventions (48). Further analysis of SDI and injury burden through health inequality assessments suggests that while the gap between wealthy and poor populations has narrowed from 1990 to 2021, SII and CI analyses indicate that the burden remains concentrated in High SDI regions and wealthier populations. Nevertheless, the burden in Low SDI countries remains concerning, potentially underestimated due to limited healthcare resources and inadequate policies (49).

The global distribution of ASDR and ASMR, along with their relationship to SDI, indicates that the highest injury burden is observed in Lesotho and South Africa (Southern Africa), Greenland (North America), and Guyana and Venezuela (South America). Among them, Greenland and Venezuela have a particularly high SIV burden, while Lesotho faces greater challenges with TI. Additionally, in Eastern Europe, Belarus and Latvia exhibit a significant UII burden. These findings align with frontier analysis, which also identifies Lithuania and the United States—both High SDI countries—as having substantial potential for improvement.

APC model analysis and heatmaps reveal that HAU-related injuries primarily affect young adults and the older adults, with TI and SIV concentrated in younger populations and UII predominantly impacting older adults. Furthermore, regions with a larger proportion of young populations, where binge drinking behaviors are more common—such as in some Low and Low-middle SDI countries—are more prone to SIV and TI. In contrast, the current severe aging trend in High-middle SDI countries corresponds to their high burden of UII. The decomposition analysis supports this pattern, showing a strong positive correlation between population aging and UII, while its impact on TI and SIV appears minimal or even negative. Population growth remains the major driver of burden changes, reflecting global demographic trends (50). APC period analysis shows a consistent decline across most regions except for Low and Low-middle SDI countries, mirroring the trend in Figure 1. Birth cohort analysis suggests that individuals born earlier face higher risks, except in Low and Low-middle SDI regions. Research by GA Roth et al. further supports this, identifying falls as the leading cause of injury-related deaths among individuals aged 70 and above (11), coinciding with increasing alcohol consumption in older adults (51), highlighting the growing impact of aging on injury burden (52), and effective health interventions for older adults can help alleviate symptoms of depression and anxiety, thereby improving quality of life and potentially reducing the injury burden indirectly caused by HAU (53). This underscores the need for region and age-specific interventions tailored to the heterogeneous burden of alcohol-related injuries across different regions and age groups. Strategies include strengthening legislation, raising the legal drinking age, and improving older adults healthcare services. Studies suggest that increasing the minimum drinking age reduces traffic injuries, suicides, and violent behaviors among young people (54–56). Additionally, fall prevention strategies for older adults should integrate alcohol consumption assessments with medication reviews (57). While population-wide interventions have proven effective, their implementation requires significant human and financial resources. Therefore, policies should be adapted based on national development levels and SDI classifications to ensure feasibility and effectiveness (58).

The BAPC model projections indicate that the HAU-related injury burden remains a significant concern globally and regionally. DALYs are expected to rise worldwide, with China and India showing increasing trends in both DALYs and ASDR. In SIV, both nations exhibit an upward trajectory, particularly India, where DALYs are projected to grow from 624,786 (95% UI: 616,470–633,103) to 747,058 (95% UI: 468,020–1,026,098). Similarly, TI-related ASDR is anticipated to rise in both countries. This part reflects the impact of policy, as regulatory measures and alcohol control programs vary across Indian states, yet most suffer from weak enforcement and implementation. At the federal level, India still lacks a comprehensive national alcohol policy (59). Moreover, Lesotho, South Africa, Venezuela (Bolivarian Republic of), the United States Virgin Islands, Congo, Equatorial Guinea, Belarus, and Latvia, identified as high-priority regions for burden reduction, are predicted to experience further increases in DALYs and ASDR, highlighting a persistently severe situation. Age-specific analysis across these countries aligns with global patterns, with young adults and the older adults bearing the highest burden. These countries facing severe challenges should establish strong alcohol control policies, such as developing a national action plan from a public health perspective, raising the legal drinking age, improving taxation policies, restricting alcohol advertising, and advancing community-level actions. At the same time, strengthening healthcare measures is also an indispensable component for alleviating the current critical situation (60).

Notably, some Deaths metrics have been included in the Supplementary materials, as their overall trends largely align with DALYs. However, certain differences exist. For instance, in the APC birth cohort effect, the older adults Deaths risk ratio is lower than that of DALYs. Similarly, decomposition analysis shows that aging has a weaker positive correlation with Deaths than with DALYs. Additionally, SDI-based regional analyses and frontier assessments reveal minor discrepancies between Deaths and DALYs across certain countries. These differences may stem from the higher proportion of younger individuals in the HAU-related injury burden, variations in data sources and quality, or the impact of public health interventions (61). Further research is required to clarify these distinctions.

Although the HAU-related injury burden appears controlled, it remains a critical issue, potentially exceeding current estimates due to unaccounted factors such as alcohol-related mental health disorders and reduced well-being. Quantifying its full short- and long-term impact may take decades. Moreover, incomplete data collection, particularly in low and middle-income countries, likely underestimates the actual burden (62). Fortunately, policy interventions have proven effective in mitigating HAU-related injuries (63). Implementing optimal national alcohol control measures could yield substantial human capital and economic benefits. However, these policies remain underutilized, with their full potential largely unrealized, further underscoring the importance of this study (64). The findings provide evidence-based guidance for countries to develop targeted interventions based on injury burden type, age distribution, and SDI level. For example, high-SDI countries like Latvia and Lithuania should focus on UII prevention, particularly among the older adults by ensuring adequate healthcare coverage for the older adults and improving their mental health, In contrast, low- and middle-SDI regions should prioritize SIV and TI reduction, especially in younger populations. Countries with severe burdens, including China, India, South Africa, Venezuela (Bolivarian Republic of), and Greenland, face urgent challenges, necessitating immediate, tailored policy actions to mitigate HAU-related injuries. Such measures may include raising the legal drinking age, strengthening road safety infrastructure and enforcement, implementing standardized alcohol control policies, and addressing the physical and mental health of young people.

Despite the advantages of the GBD 2021 dataset in providing standardized and comparable estimates across countries and over time, several limitations must be acknowledged. First, the quality of data varies considerably across regions. Many low- and middle-income countries lack reliable injury surveillance systems or comprehensive vital registration, which may lead to underestimation or misclassification of alcohol-related injuries. Second, the GBD relies on statistical modeling and interpolation methods to fill data gaps. While these methods are indispensable, they may obscure true regional heterogeneity and introduce uncertainty, particularly in countries where input data are sparse or of poor quality. Third, the modeling process may not fully capture differences in local culture, behaviors, or healthcare systems. Finally, predictive methods (e.g., BAPC) extend historical trends into the future but cannot account for unforeseen shocks, such as sudden policy changes or emerging health threats. These limitations suggest that our findings should be interpreted with caution and supplemented with national-level data whenever available.

## Conclusion

This study provides the first comprehensive global and regional assessment of HAU-related injuries, integrating SIV, TI, and UII across 204 countries from 1990 to 2021 with projections to 2030. Despite global declines in ASDR and ASMR, absolute numbers of deaths and DALYs continue to rise, with SIV and TI burdens increasing in low- and middle-SDI regions and UII persisting in high-SDI countries due to aging populations. Decomposition and APC analyses confirm that young adults are disproportionately affected by SIV and TI, while older adults face high UII burdens. Frontier and inequality analyses reveal substantial room for improvement across all SDI levels. These findings underscore the urgent need for targeted, context-specific interventions, including stricter alcohol policies for youth, enhanced healthcare and fall-prevention programs for the older adults, stronger road safety measures, and standardized alcohol control strategies. Coordinated global and national actions, tailored to regional development levels and demographic profiles, are essential to reduce preventable injuries and improve population health.

## Data Availability

The datasets presented in this study can be found in online repositories. The names of the repository/repositories and accession number(s) can be found in the article/Supplementary material.

## Supplementary material

Supplementary Figure S1 The relationship between ASMR and SDI for HAU-related Injuries **(A)**, SIV **(B)**, TI **(C)**, and UII **(D)** across 204 countries in 2021. HAU, high alcohol use; SIV, self-harm and interpersonal violence; TI, transport injuries; UII, unintentional injuries; ASMR, age-standardized mortality rate; SDI, socio-demographic index.

Supplementary Figure S2 The geographic distribution of ASMR for HAU-related Injuries, SIV, TI, and UII across 204 countries and regions in 2021 [**(A)** Injuries, **(B)** SIV, **(C)** TI, **(D)** UII]. Abbreviations: HAU, high alcohol use; SIV, self-harm and interpersonal violence; TI, transport injuries; UII, unintentional injuries; ASMR, age-standardized mortality rate.

Supplementary Figure S3 The effects of age, period, and birth cohort on ASMR for HAU-related Injuries, SIV, TI, and UII based on the APC model. Panels **(A–C)** represent the age, period, and cohort effects for Injuries, **(D–F)** for SIV, **(G–I)** for TI, and **(J–L)** for UII. APC, annual percentage change; HAU, high alcohol use; SIV, self-harm and interpersonal violence; TI, transport injuries; UII, unintentional injuries; ASMR, age-standardized mortality rate.

Supplementary Figure S4 The relative contributions of aging, population dynamics, and epidemiological changes to ASMR variations in HAU-related injuries, SIV, TI, and UII from 1990 to 2021, with comparisons between global data and five SDI regions. Black dots indicate the overall ASMR change during this period. Panels **(A–D)** corresponds to injuries, SIV, TI, and UII, respectively. HAU, high alcohol use; SIV, self-harm and interpersonal violence; TI, transport injuries; UII, unintentional injuries; ASMR, age-standardized mortality rate.

Supplementary Figure S5 The Slope Index of Inequality (SII) and Concentration Index (CI) of ASMR for HAU-related injuries, SIV, TI, and UII in 1990 and 2021 worldwide. **(A)** Represents the SII for Injuries, illustrating the relationship between SDI and ASIR across countries, with data point size proportional to population. **(B)** Displays the CI for Injuries, quantifying relative health disparities by measuring the area under the Lorenz curve, aligning ASIR distribution with the SDI-based population distribution. **(C, D)** show the SII and CI for SIV, respectively, while **(E, F)** present the same for TI. Similarly, **(G, H)** depict the SII and CI for UII. SII, slope index of inequality; CI, concentration index; HAU, high alcohol use; SIV, self-harm and interpersonal violence; TI, transport injuries; UII, unintentional injuries; ASMR, age-standardized mortality rate; SDI, socio-demographic index.

Supplementary Figure S6 Frontier analysis of ASMR for HAU-related Injuries, SIV, TI, and UII based on SDI in 2021. The black boundary line represents the theoretically achievable ASDR given SDI, while each point denotes the actual ASDR of a specific country or region. Color gradients indicate data collection years, ranging from dark blue (1990) to light blue (2021). The 15 countries with the largest effective disparities—defined as the gap between observed and achievable ASDR—are marked in black. The five countries with the smallest disparities in Low-SDI regions (<0.50) are highlighted in blue, whereas the country with the highest disparity in High-SDI regions (>0.85) is marked in red. Panels **(A, B)** corresponds to injuries, **(C, D)** to SIV, **(E, F)** to TI, and **(G, H)** to UII. HAU, high alcohol use; SIV, self-harm and interpersonal violence; TI, transport injuries; UII, unintentional injuries; ASMR, age-standardized mortality rate.

Supplementary Figure S7 Age distribution of ASDR in the five countries and regions with the greatest potential for burden reduction in HAU-related injuries, SIV, TI, and UII in 2021. Panels **(A–E)** correspond to Injuries, **(F, J)** to SIV, **(K–O)** to TI, and **(P–T)** to UII. HAU, high alcohol use; SIV, self-harm and interpersonal violence; TI, transport injuries; UII, unintentional injuries; ASDR, age-standardized disability-adjusted life years rate.

Supplementary Figure S8 Projected DALYs and ASDR from 2022 to 2030 for HAU-related injuries, SIV, TI, and UII in the five countries and regions with the greatest potential for burden reduction. Panels **(A–E)** corresponds to Injuries, **(F, J)** to SIV, **(K–O)** to TI, and **(P–T)** to UII. HAU, high alcohol use; SIV, self-harm and interpersonal violence; TI, transport injuries; UII, unintentional injuries; DALYs, disability-adjusted life years; ASDR, age-standardized disability-adjusted life years rate.
