# Supplementary figures and images for: Global burden and trends of high alcohol use-related injuries from 1990 to 2030: a comprehensive assessment of self-harm and interpersonal violence, transport injuries, and unintentional injuries using global burden of disease 2021
Source: Front Public Health. 2025 Oct 16;13:1675607. doi: 10.3389/fpubh.2025.1675607 (PMC12571873; doi:10.3389/fpubh.2025.1675607)

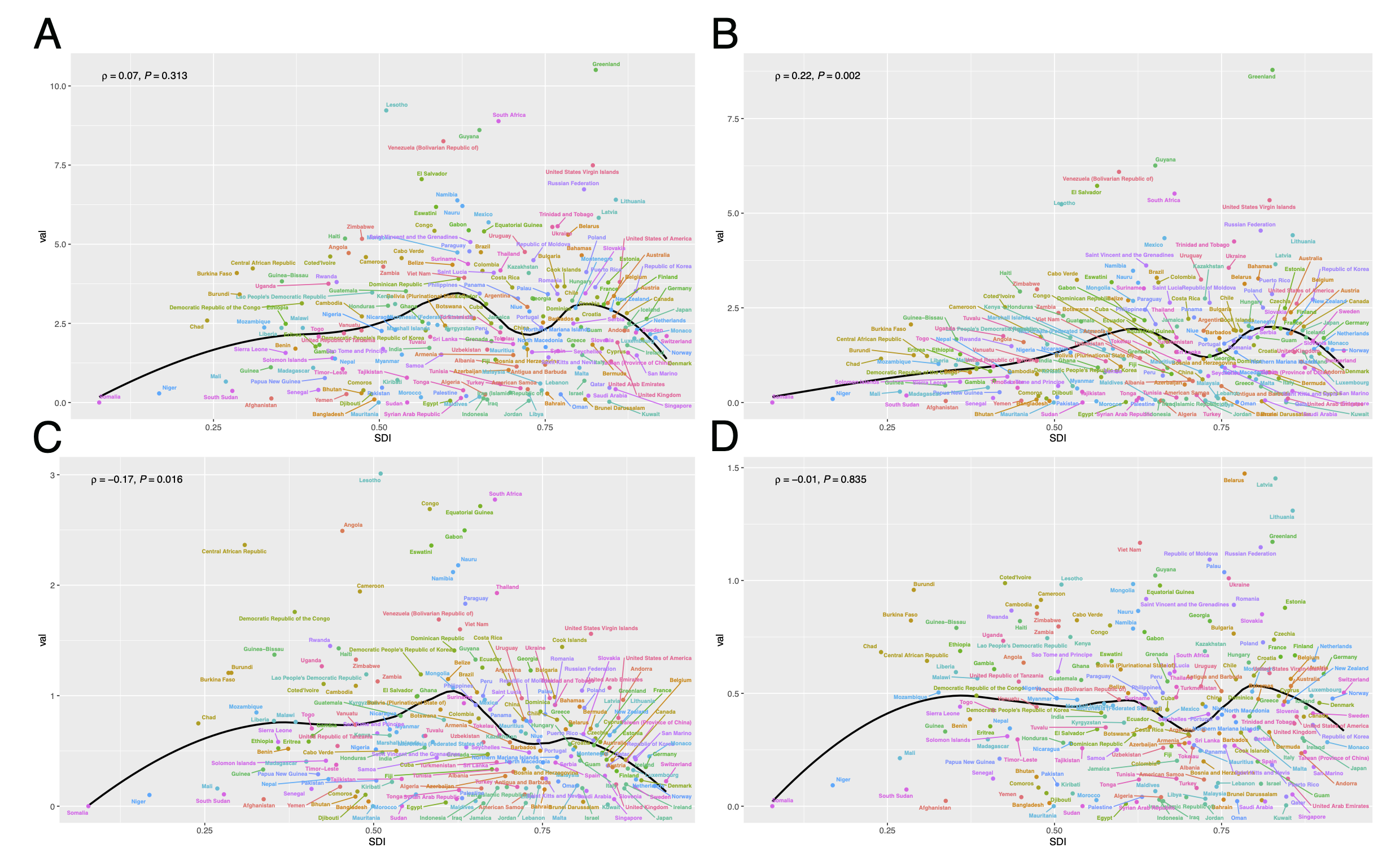

Supplement: Supplementary Figure S1 — The relationship between ASMR and SDI for HAU-related Injuries (A), SIV (B), TI (C), and UII (D) across 204 countries in 2021. HAU, high alcohol use; SIV, self-harm and interpersonal violence; TI, transport injuries; UII, unintentional injuries; ASMR, age-standardized mortality rate; SDI, socio-demographic index. [file Image_1.tiff]

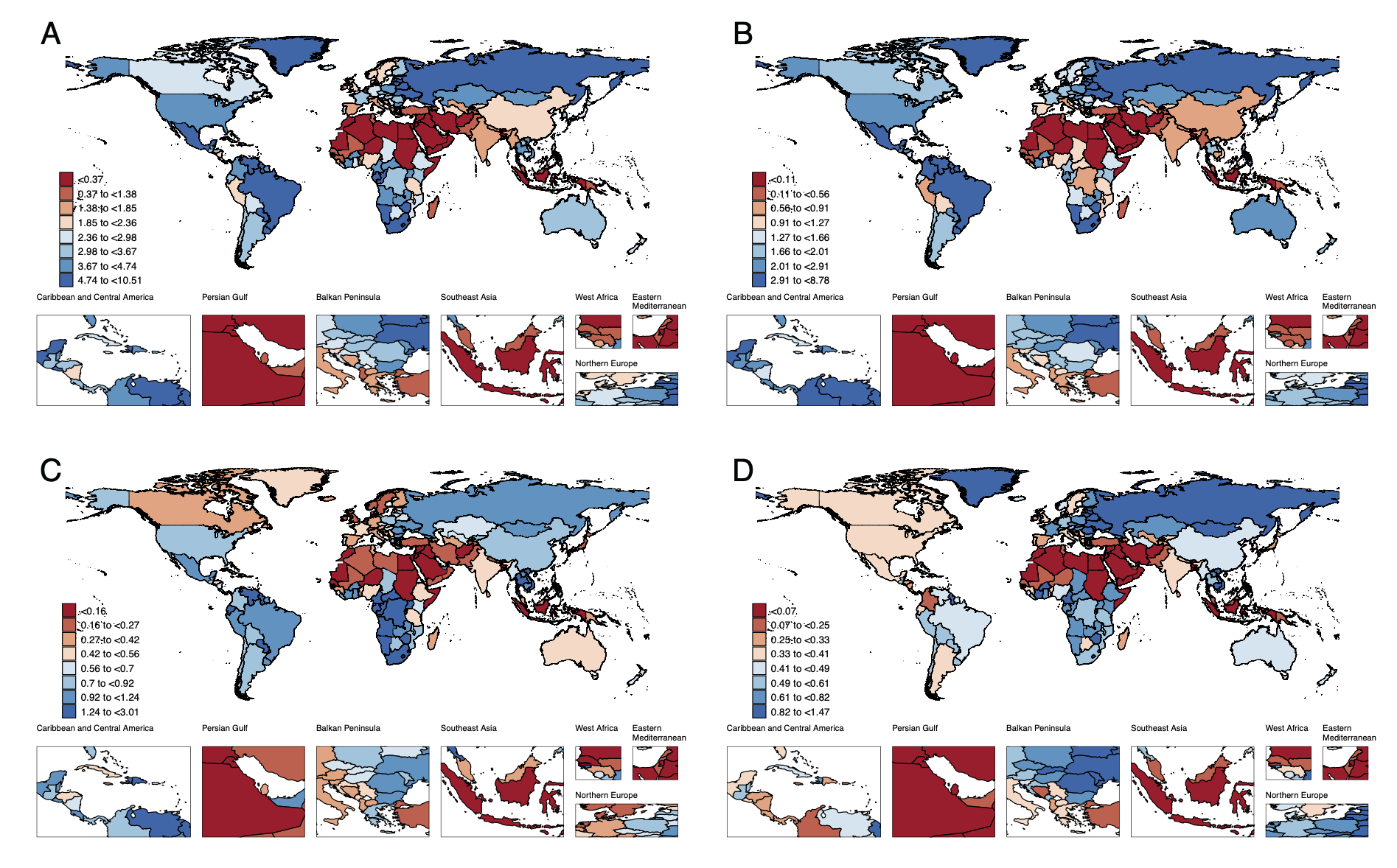

Supplement: Supplementary Figure S2 — The geographic distribution of ASMR for HAU-related Injuries, SIV, TI, and UII across 204 countries and regions in 2021 [(A) Injuries, (B) SIV, (C) TI, (D) UII]. Abbreviations: HAU, high alcohol use; SIV, self-harm and interpersonal violence; TI, transport injuries; UII, unintentional injuries; ASMR, age-standardized mortality rate. [file Image_2.tiff]

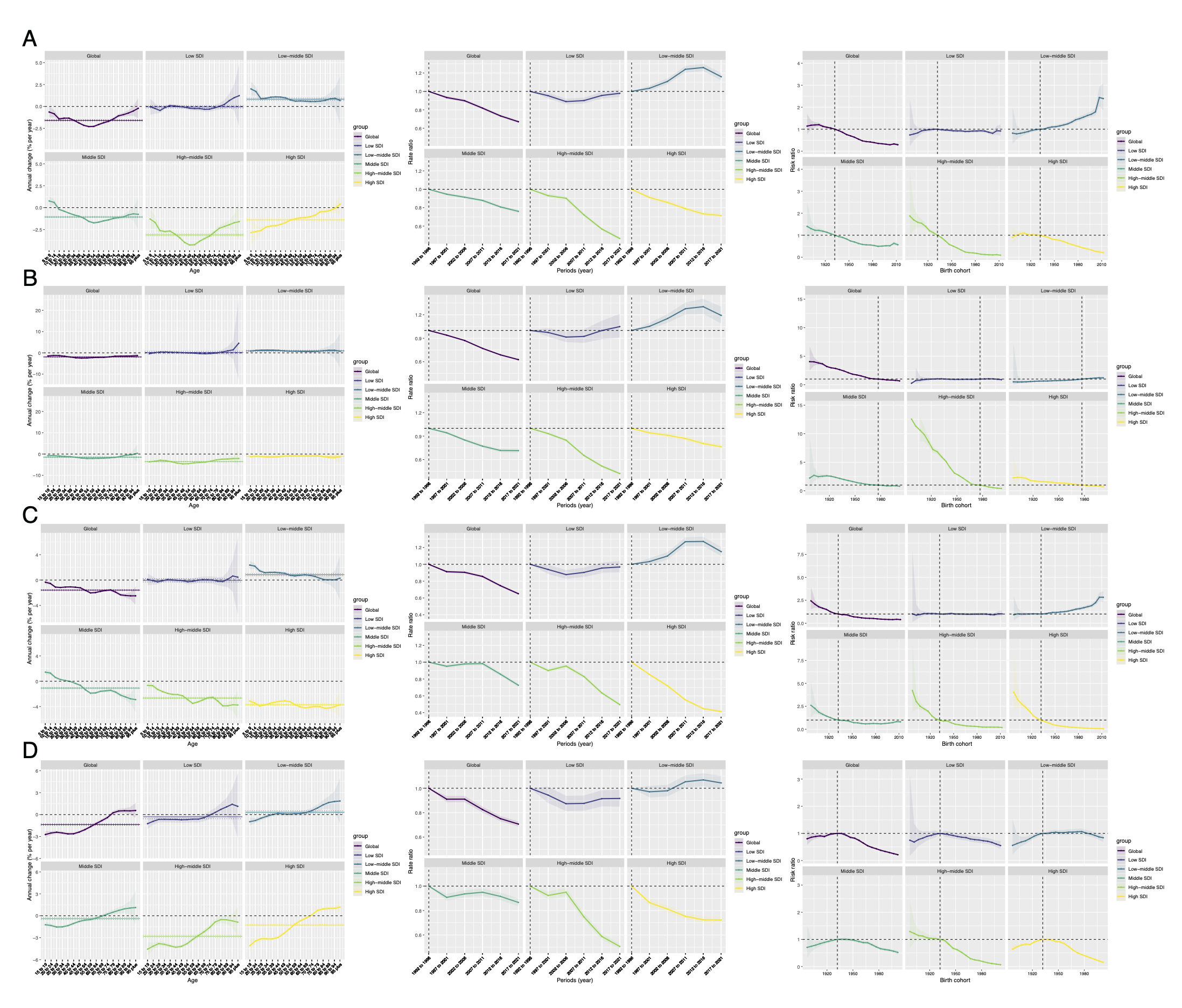

Supplement: Supplementary Figure S3 — The effects of age, period, and birth cohort on ASMR for HAU-related Injuries, SIV, TI, and UII based on the APC model. Panels (A–C) represent the age, period, and cohort effects for Injuries, (D–F) for SIV, (G–I) for TI, and (J–L) for UII. APC, annual percentage change; HAU, high alcohol use; SIV, self-harm and interpersonal violence; TI, transport injuries; UII, unintentional injuries; ASMR, age-standardized mortality rate. [file Image_3.tiff]

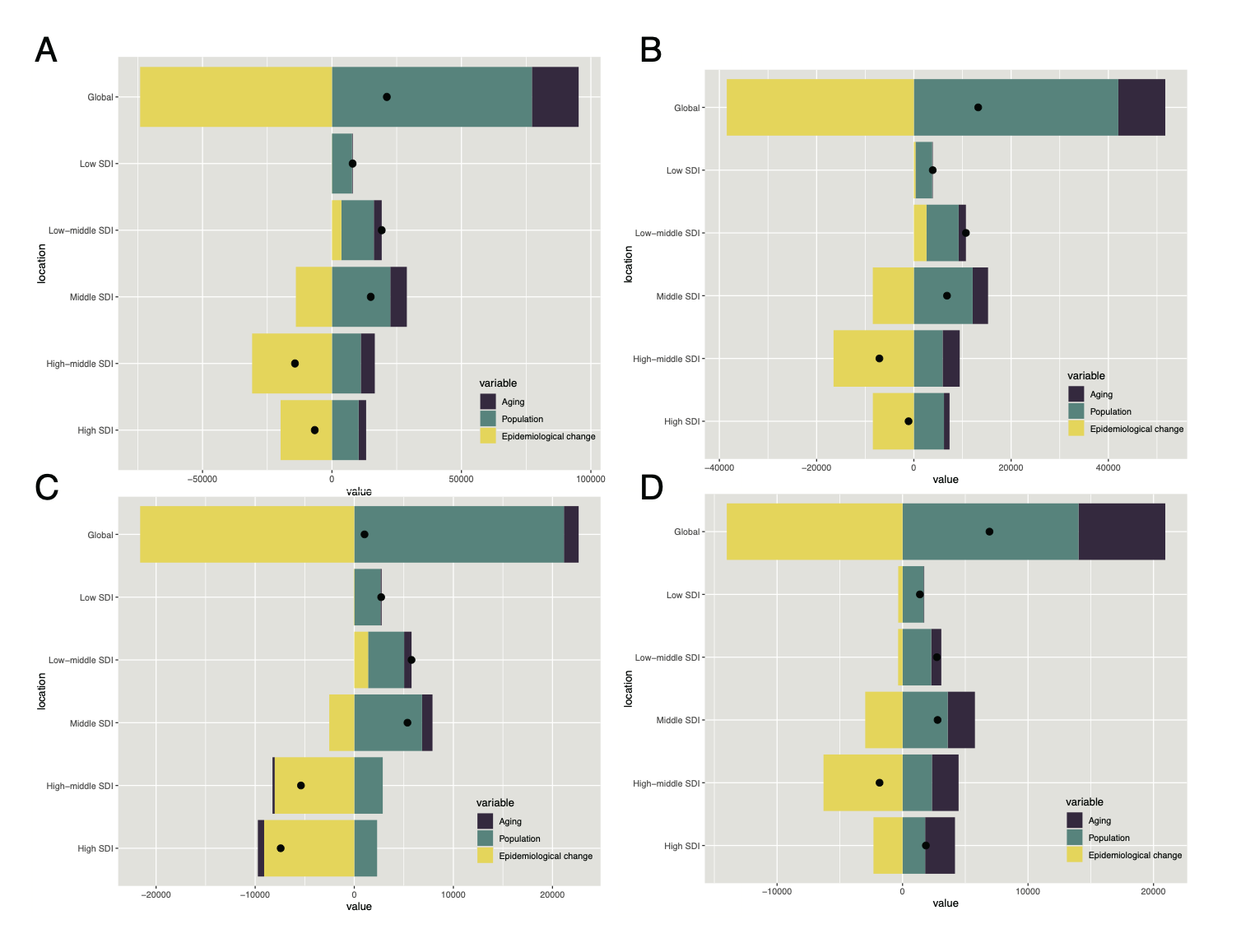

Supplement: Supplementary Figure S4 — The relative contributions of aging, population dynamics, and epidemiological changes to ASMR variations in HAU-related injuries, SIV, TI, and UII from 1990 to 2021, with comparisons between global data and five SDI regions. Black dots indicate the overall ASMR change during this period. Panels (A–D) corresponds to injuries, SIV, TI, and UII, respectively. HAU, high alcohol use; SIV, self-harm and interpersonal violence; TI, transport injuries; UII, unintentional injuries; ASMR, age-standardized mortality rate. [file Image_4.tiff]

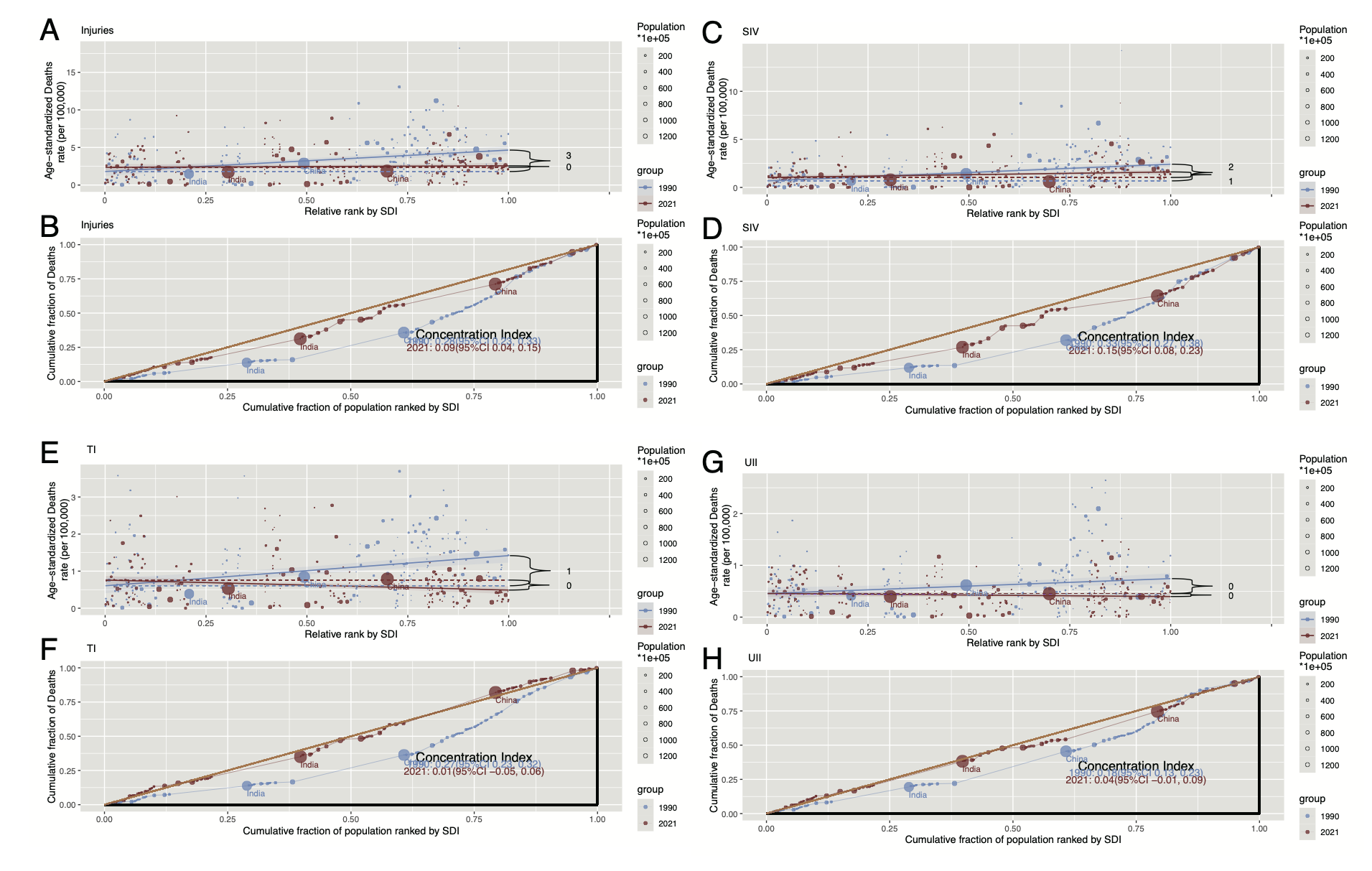

Supplement: Supplementary Figure S5 — The Slope Index of Inequality (SII) and Concentration Index (CI) of ASMR for HAU-related injuries, SIV, TI, and UII in 1990 and 2021 worldwide. (A) Represents the SII for Injuries, illustrating the relationship between SDI and ASIR across countries, with data point size proportional to population. (B) Displays the CI for Injuries, quantifying relative health disparities by measuring the area under the Lorenz curve, aligning ASIR distribution with the SDI-based population distribution. (C, D) show the SII and CI for SIV, respectively, while (E, F) present the same for TI. Similarly, (G, H) depict the SII and CI for UII. SII, slope index of inequality; CI, concentration index; HAU, high alcohol use; SIV, self-harm and interpersonal violence; TI, transport injuries; UII, unintentional injuries; ASMR, age-standardized mortality rate; SDI, socio-demographic index. [file Image_5.tiff]

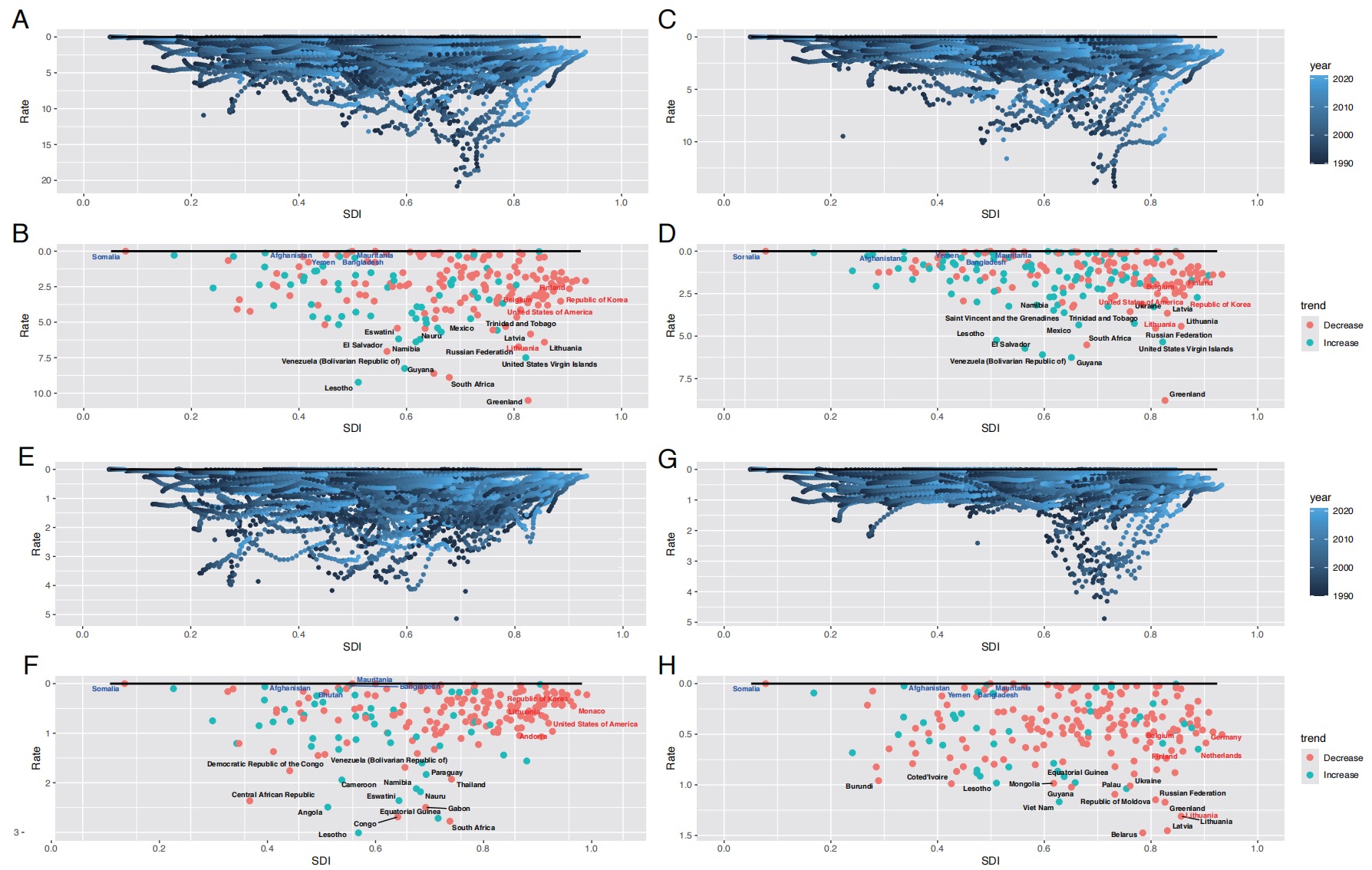

Supplement: Supplementary Figure S6 — Frontier analysis of ASMR for HAU-related Injuries, SIV, TI, and UII based on SDI in 2021. The black boundary line represents the theoretically achievable ASDR given SDI, while each point denotes the actual ASDR of a specific country or region. Color gradients indicate data collection years, ranging from dark blue (1990) to light blue (2021). The 15 countries with the largest effective disparities—defined as the gap between observed and achievable ASDR—are marked in black. The five countries with the smallest disparities in Low-SDI regions (<0.50) are highlighted in blue, whereas the country with the highest disparity in High-SDI regions (>0.85) is marked in red. Panels (A, B) corresponds to injuries, (C, D) to SIV, (E, F) to TI, and (G, H) to UII. HAU, high alcohol use; SIV, self-harm and interpersonal violence; TI, transport injuries; UII, unintentional injuries; ASMR, age-standardized mortality rate. [file Image_6.tiff]

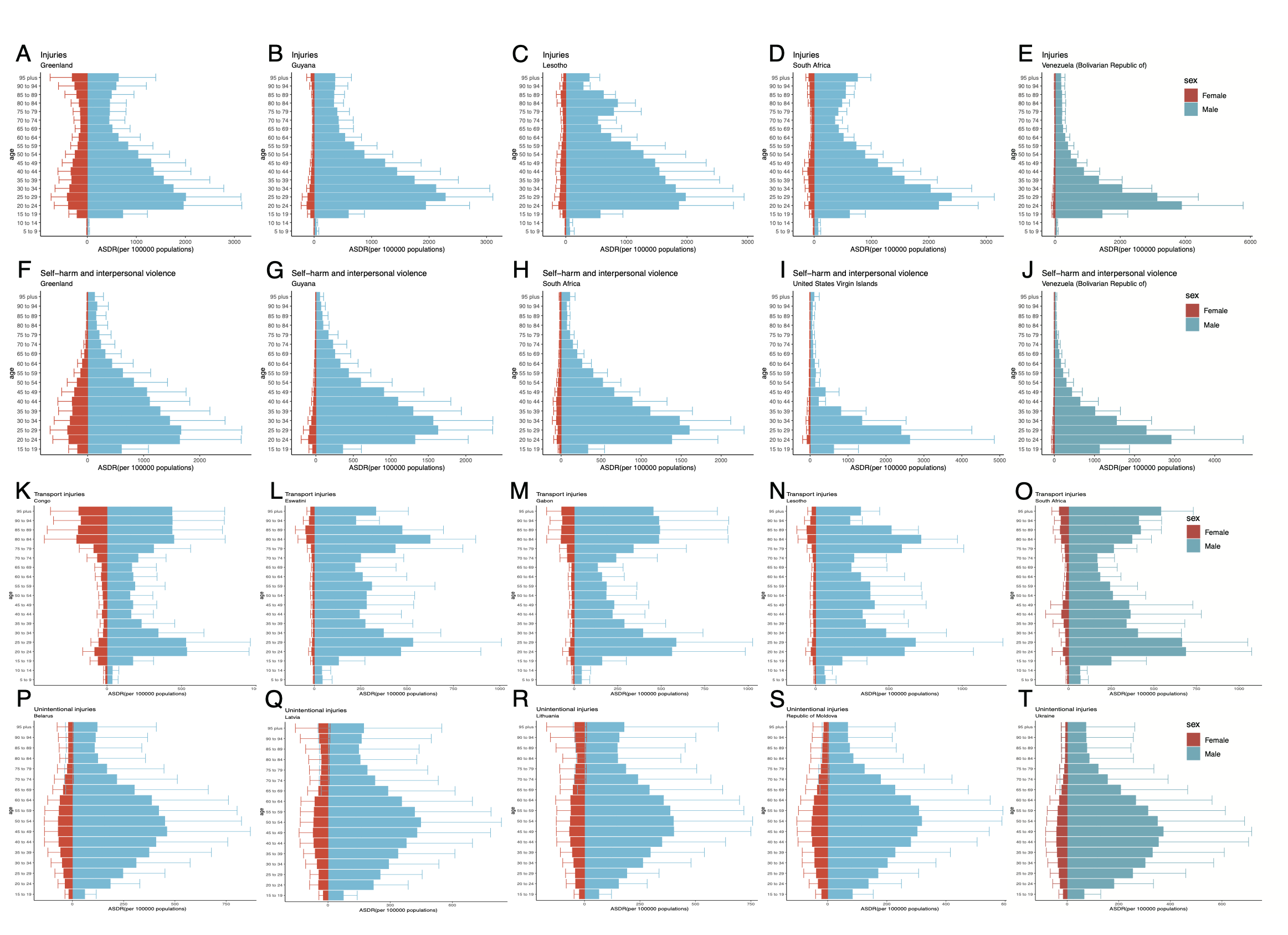

Supplement: Supplementary Figure S7 — Age distribution of ASDR in the five countries and regions with the greatest potential for burden reduction in HAU-related injuries, SIV, TI, and UII in 2021. Panels (A–E) correspond to Injuries, (F, J) to SIV, (K–O) to TI, and (P–T) to UII. HAU, high alcohol use; SIV, self-harm and interpersonal violence; TI, transport injuries; UII, unintentional injuries; ASDR, age-standardized disability-adjusted life years rate. [file Image_7.tiff]

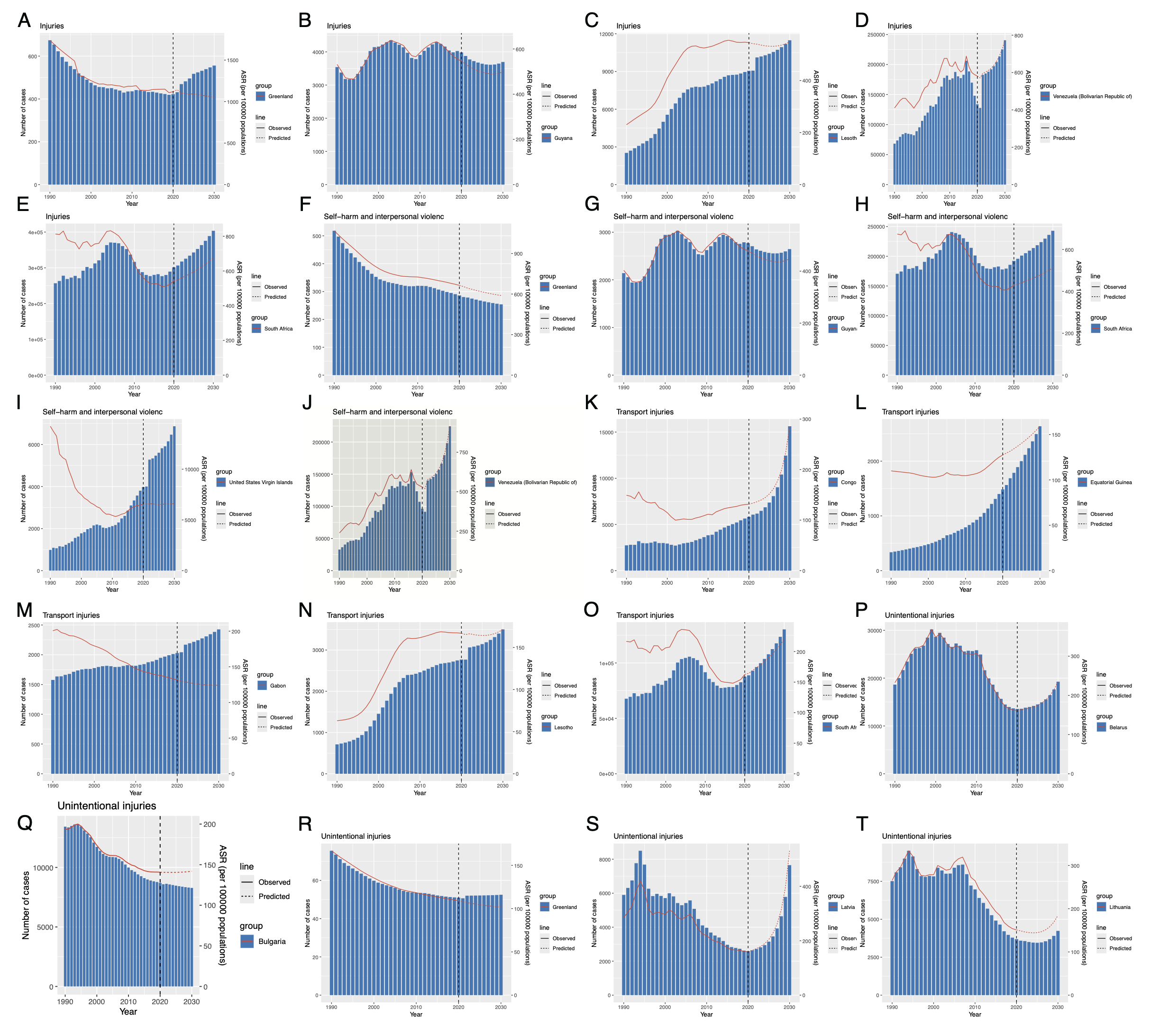

Supplement: Supplementary Figure S8 — Projected DALYs and ASDR from 2022 to 2030 for HAU-related injuries, SIV, TI, and UII in the five countries and regions with the greatest potential for burden reduction. Panels (A–E) corresponds to Injuries, (F, J) to SIV, (K–O) to TI, and (P–T) to UII. HAU, high alcohol use; SIV, self-harm and interpersonal violence; TI, transport injuries; UII, unintentional injuries; DALYs, disability-adjusted life years; ASDR, age-standardized disability-adjusted life years rate. [file Image_8.tiff]
